# Integrative single-cell and spatial transcriptomic analyses identify *LMX1B* as a tumor suppressor orchestrating the *GDF15*–*ATP4B* axis in renal cell carcinoma

**DOI:** 10.1016/j.tranon.2026.103049

**Published:** 2026-09-18

**Authors:** Yu Liu, Liang Song, Yuankang Feng, Songxin Zhang, Xu Han, Youzhi Tan, Ningyang Li, Yingjin Wang, Shaoliang Sun, Jingwei Gao, Long Zhang, Zhankui Jia, Jinjian Yang

**Affiliations:** a Department of Urology, The First Affiliated Hospital of Zhengzhou University, No. 1 Jianshe East Road, Zhengzhou, Henan, 450052, China; b Department of Urology, Tianjin Institute of Urology, The Second Hospital of Tianjin Medical University, Tianjin, 300211, China

**Keywords:** LMX1B, Single-cell and spatial transcriptomics, Tumor microenvironment, Mitochondrial apoptosis, GDF15

## Abstract

**Background:**

Renal cell carcinoma (RCC) is characterized by profound inter- and intratumoral heterogeneity and extensive tumor microenvironment (TME) remodeling, yet the spatially resolved cellular programs and intercellular communication networks orchestrating malignant progression remain poorly defined. *LMX1B*, a LIM homeodomain transcription factor essential for kidney morphogenesis, has not been previously investigated in renal carcinogenesis.

**Methods:**

We integrated single-cell RNA sequencing (scRNA-seq) and spatial transcriptomics (ST) across three independent RCC cohorts, complemented by hierarchical differential weighted gene co-expression network analysis (hdWGCNA), CellChat-based ligand-receptor interaction analysis, RCTD spatial deconvolution, and mistyR colocalization analysis. Multi-platform validation included qRT-PCR, western blot, TCGA and CCLE database mining, chromatin immunoprecipitation, dual-luciferase reporter assays, and functional studies *in vitro* and in subcutaneous xenograft models.

**Results:**

ScRNA-seq and ST analyses revealed that *LMX1B* is predominantly expressed in epithelial cells and is markedly downregulated in tumor-derived epithelial populations compared to normal counterparts, with reduced expression correlating with advanced tumor grade and lymphatic metastasis. *LMX1B*-associated epithelial cells (*LMX1B*-Epi) function as central signaling hubs within the TME, engaging in extensive bidirectional communication with fibroblasts, endothelial cells, myeloid cells, and T/NK cells through *VEGFA*-, *SPP1*-, and *GDF15*-mediated pathways. Spatial deconvolution demonstrated that *LMX1B*-Epi occupy specialized tissue niches distinct from *LMX1B*-negative epithelial cells. HdWGCNA identified five co-expression modules enriched for stress response, metabolism, and mitochondrial ATP synthesis, all exhibiting significant differential activation between normal and tumor *LMX1B*-Epi. Functionally, *LMX1B* overexpression suppressed RCC cell proliferation, migration, and invasion. Mechanistically, *LMX1B* directly binds to the *GDF15* promoter to transcriptionally activate its expression. *GDF15*, in turn, positively regulates *ATP4B* and induces mitochondrial apoptosis through ROS-mediated mitochondrial membrane potential depolarization and Bax/Bcl-2-caspase-3 cascade activation. Rescue experiments confirmed that *ATP4B* overexpression reverses the oncogenic effects of *GDF15* knockdown both *in vitro* and *in vivo*.

**Conclusions:**

Our integrative multi-omic and functional analyses establish the *LMX1B*–*GDF15*–*ATP4B* axis as a critical tumor-suppressive cascade in RCC. These findings illuminate how a developmentally essential transcription factor governs TME organization and mitochondrial homeostasis in renal malignancy, and identify *LMX1B* as a promising prognostic biomarker and therapeutic target for RCC.

## Highlights

• Integrative single-cell and spatial transcriptomic analyses identify LMX1B as a previously unrecognized tumor suppressor in renal cell carcinoma.
• LMX1B-associated epithelial cells function as central signaling hubs within the tumor microenvironment, engaging in spatially organized bidirectional communication with stromal and immune compartments.
• LMX1B transcriptionally activates GDF15 through direct promoter binding, establishing the LMX1B–GDF15–ATP4B axis as a critical tumor-suppressive regulatory cascade.
• GDF15 induces mitochondrial apoptosis via ROS-mediated membrane potential depolarization and Bax/Bcl-2-caspase-3 cascade activation in RCC cells.
• These findings identify LMX1B as a promising prognostic biomarker and provide multiple druggable nodes for precision therapeutic intervention in renal malignancy.

## Graphical abstract

**Figure:**
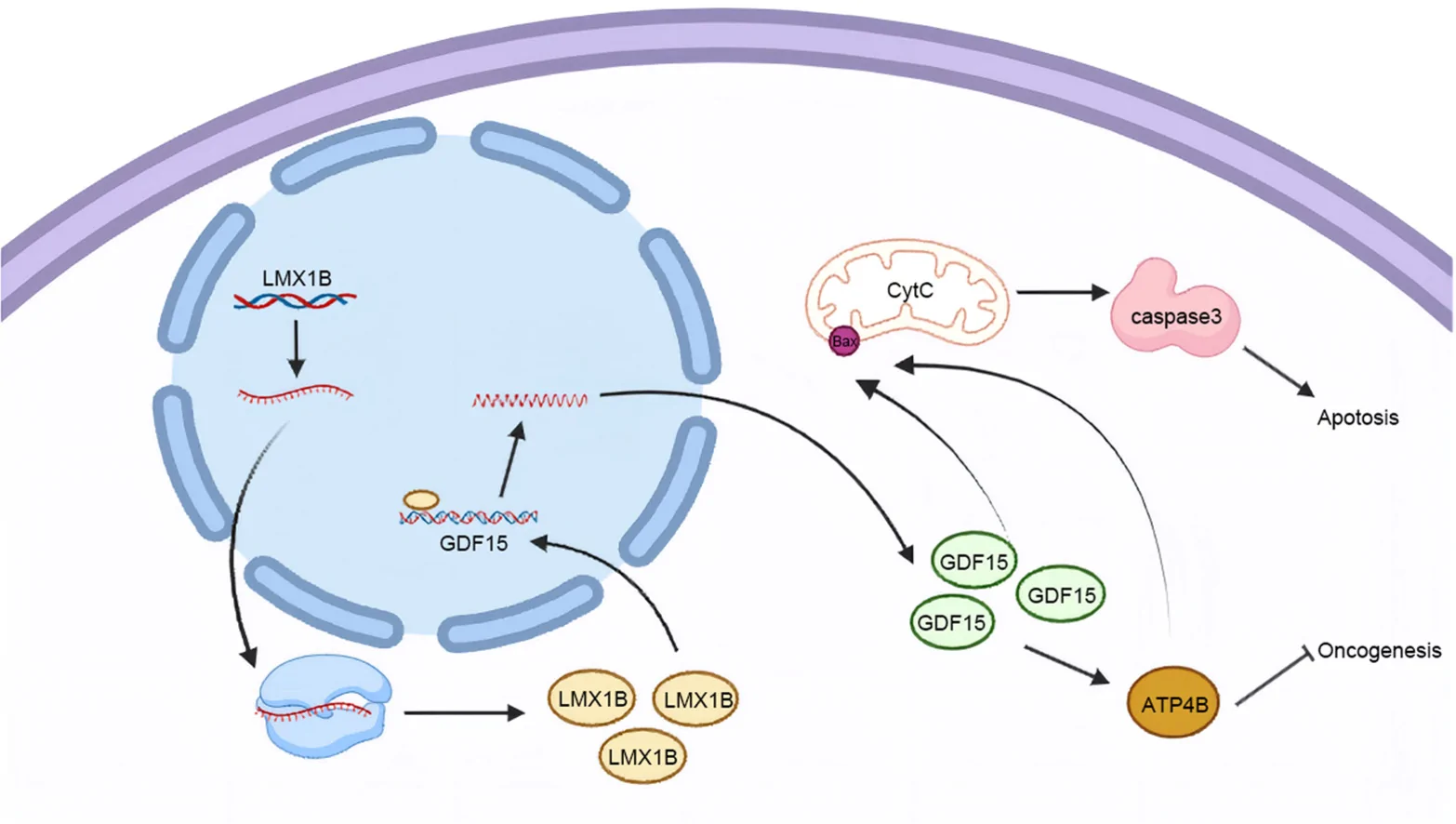

## Introduction

Renal cell carcinoma (RCC) represents a paradigm of inter- and intratumoral heterogeneity, posing formidable challenges to therapeutic intervention. As the ninth most prevalent malignancy among males and fourteenth among females worldwide, RCC accounts for over 170,000 cancer-related deaths annually, ranking as the 16th leading contributor to global cancer mortality. Clear cell RCC (ccRCC), constituting 70–75% of all renal malignancies [1], is characterized by profound genomic instability [2], metabolic reprogramming [3], and extensive remodeling of the tumor microenvironment (TME) [4]. Despite the advent of immune checkpoint inhibitors and tyrosine kinase inhibitors that have transformed the therapeutic landscape for advanced disease, the majority of patients ultimately experience disease progression, underscoring an urgent need to decipher the molecular underpinnings driving RCC initiation and malignant evolution [5]. The intricate interplay between cancer cells and their surrounding stromal, immune, and vascular compartments within the TME has emerged as a critical determinant of tumor behavior, yet the spatially resolved cellular hierarchies and intercellular communication networks governing RCC progression remain incompletely understood [6].

Recent advances in single-cell RNA sequencing (scRNA-seq) and spatial transcriptomics (ST) have revolutionized our capacity to dissect tumor ecosystems with unprecedented resolution [7]. These complementary technologies enable the deconvolution of cellular heterogeneity at the transcriptomic level while preserving the spatial architecture and contextual relationships that define tissue organization. In solid tumors, scRNA-seq has uncovered rare cancer cell subpopulations harboring distinct genetic and phenotypic programs that drive treatment resistance and metastatic dissemination, whereas ST has revealed spatially distinct molecular niches within tumors that harbor unique therapeutic vulnerabilities. The integration of these multi-omic platforms offers a powerful framework to reconstruct intercellular communication networks, identify context-dependent biomarkers, and elucidate the spatially organized molecular programs that orchestrate tumor progression—insights that are unattainable through bulk transcriptomic analyses alone [8].

The TME of RCC is composed of a complex milieu of malignant epithelial cells, fibroblasts, endothelial cells, and diverse immune populations, each contributing to a dynamic ecosystem that fosters tumor growth, immune evasion, and therapeutic resistance [9]. Emerging evidence from single-cell atlases of kidney cancer has highlighted substantial alterations in TME composition between normal adjacent tissue and tumor tissue, with distinct spatial distributions of immune and stromal cell types correlating with clinicopathological features. However, the identification of epithelial cell-intrinsic transcriptional programs that serve as nodal regulators of TME organization and the elucidation of their spatially restricted interactions with neighboring cell types remain critical gaps in our understanding of RCC biology [10]. Transcription factors (TFs), as master regulators of gene expression networks, occupy a privileged position in this regulatory hierarchy, yet the vast majority of TFs with established developmental functions remain unexplored in the context of renal malignancy [11].

*LMX1B*, a member of the LIM homeodomain protein family, is a pivotal determinant of dorsal limb fate and kidney morphogenesis during vertebrate development [12]. Structurally characterized by tandem LIM domains that mediate protein-protein interactions and a homeodomain that governs DNA binding and transcriptional activation [13], *LMX1B* orchestrates the dorsoventral patterning of limbs and the initial morphogenesis of multiple tissues including kidneys, eyes, and brain. Germline mutations in *LMX1B* are causally linked to nail-patella syndrome and a spectrum of renal developmental disorders, underscoring its non-redundant role in nephrogenesis [14]. Despite its well-documented functions in embryonic kidney development and adult renal physiology, the involvement of *LMX1B* in renal carcinogenesis has remained entirely unexplored. This knowledge gap is particularly striking given the emerging recognition that developmental TFs frequently undergo dysregulation during tumorigenesis, where they can function as either oncogenic drivers or tumor suppressors depending on cellular context [[15], [16], [17]].

Growth differentiation factor 15 (*GDF15*), a divergent member of the transforming growth factor-β superfamily, exhibits pleiotropic and context-dependent functions in cancer biology [18]. While *GDF15* has been implicated in promoting tumor progression in prostate and pancreatic carcinomas through diverse mechanisms, it exerts tumor-suppressive effects in colorectal cancer, highlighting the necessity of investigating its role in a tissue-specific manner [[19], [20], [21], [22]]. The downstream mediators of *GDF15* signaling in RCC remain undefined, although its capacity to modulate cellular stress responses and mitochondrial homeostasis suggests potential intersections with metabolic pathways central to renal malignancy. *ATP4B*, encoding the β-subunit of the gastric H+/K+-ATPase, has recently been identified as a putative tumor suppressor in gastric cancer [23], where its restoration inhibits proliferation and induces apoptosis. Intriguingly, emerging evidence has linked *ATP4B* to RCC prognosis [24], yet the upstream regulatory mechanisms governing its expression and its functional contribution to renal carcinogenesis remain unknown [25].

In this study, we integrated scRNA-seq and spatial transcriptomic analyses across three independent RCC cohorts with multidimensional functional validation to systematically investigate the role of *LMX1B* in RCC pathogenesis. Our analyses revealed that *LMX1B* is predominantly expressed in epithelial cells and is markedly downregulated during malignant transformation, with its expression inversely correlating with tumor grade and lymphatic metastasis. Through hierarchical differential weighted gene co-expression network analysis (hdWGCNA) and spatial deconvolution, we demonstrate that *LMX1B*-associated epithelial cells occupy specialized niches within the TME and engage in spatially organized intercellular communication networks. Mechanistically, we establish that *LMX1B* transcriptionally activates *GDF15* through direct promoter binding, and that *GDF15* subsequently upregulates *ATP4B* to induce mitochondrial apoptosis via ROS-mediated depolarization and Bax/Bcl-2-caspase-3 cascade activation. Collectively, our findings delineate the *LMX1B*–*GDF15*–*ATP4B* axis as a critical regulatory cascade suppressing RCC progression, and identify *LMX1B* as a promising prognostic biomarker and therapeutic target for this lethal malignancy.

## Material and methods

### Data collection

Single-cell RNA sequencing (scRNA-seq) data for renal cell carcinoma (RCC) were obtained from three independent public datasets: GSE210038(n=9 samples), GSE224630(n=7 samples), and GSE242299(n=31 samples). Spatial transcriptomics data were derived from the corresponding spatial samples (ffpe_c_2, ffpe_c_3, ffpe_c_7, and ffpe_c_10) for spatial validation. Bulk transcriptomic profiles were obtained from UCSC Xena [[26], [27], [28]], including TCGA-KIRC (Kidney Renal Clear Cell Carcinoma), with the additional validation dataset GSE12008 downloaded from the Gene Expression Omnibus (GEO). The Cancer Cell Line Encyclopedia (CCLE) database was queried for *LMX1B* mRNA expression across RCC cell lines. The JASPAR database was employed for the prediction of transcription factor binding events [29^,^ 30]. The National Center for Biotechnology Information (NCBI) Reference Sequence database was utilized for chromosomal localization and promoter architecture analysis of target genes.

### Single-cell transcriptome analysis and marker gene identification

Raw single-cell transcriptomic profiles were analyzed within the Seurat environment (v4.3.0). Quality-control procedures excluded cells containing fewer than 500 detected transcripts as well as cells exhibiting mitochondrial transcript proportions greater than 15%. Artificial multiplets were subsequently identified and eliminated using DoubletFinder (v2.0.4). To reduce technical variability among samples, data integration was carried out with the Harmony algorithm. Genes displaying the highest expression variability across cells were ranked, and the 2,000 most variable features were retained for downstream analyses. Principal component decomposition was then performed, and clustering was generated using the first 50 principal components. Cellular distributions were projected into a two-dimensional space using Uniform Manifold Approximation and Projection (UMAP). Cell identities were assigned according to established lineage-associated marker genes. Cluster-enriched transcripts were determined using the FindAllMarkers function (min.pct = 0.25, logfc.threshold = 0.25, only.pos = TRUE). Statistical inference relied on the Wilcoxon rank-sum approach, and resulting P values were corrected for multiple comparisons using the Benjamini–Hochberg false discovery rate procedure.

### Intercellular signaling analysis

Communication events among cellular populations were investigated with CellChat (v2.2.0) using single-cell transcriptomic profiles. Ligand–receptor associations were annotated according to the CellChatDB.human resource. The probability of signaling exchange between distinct cell groups was estimated from the expression patterns of interacting molecules, and interactions satisfying a significance criterion of P < 0.05 were retained for subsequent network analyses.

### hdWGCNA

To explore the transcriptional programs characterizing *LMX1B*-associated epithelial cells, hdWGCNA was applied to malignant epithelial cells. Co-expression networks were established, and gene modules were detected via topological overlap analysis. Modules showing the strongest association with the *LMX1B*-associated phenotype were identified through module-trait correlation. Genes in these modules were ranked according to their module membership (KME), and the top hub genes were designated as *LMX1B*-associated hub genes for subsequent analyses.

### Spatial transcriptomic data analysis

Spatial transcriptomic datasets were analyzed with Seurat (v4.3.0). Low-quality capture locations expressing fewer than 200 genes, together with genes detected in fewer than three locations, were excluded from further analysis. Variance stabilization and normalization were carried out using SCTransform. Cross-sample integration was achieved through the Seurat workflow incorporating SelectIntegrationFeatures, PrepSCTIntegration, FindIntegrationAnchors, and IntegrateData. Principal component analysis was performed using the top 30 principal components, followed by graph-based clustering. Spatial domains were annotated according to histopathological morphology observed in hematoxylin–eosin sections and cluster-enriched genes. Spatial distributions of clusters and genes were displayed using SpatialDimPlot and SpatialFeaturePlot. To estimate cellular composition within each spatial location, Robust Cell Type Decomposition (RCTD) was applied in full-mode, leveraging single-cell transcriptomic references. Differential markers for individual cell populations were identified with FindAllMarkers using positive log2 fold-change criteria. Interactions among spatially distributed cell populations were subsequently evaluated using MISTy (mistyR v1.16.0) [31].

### Bioinformatic analysis of bulk transcriptomic data

Disparity analysis, volcano plotting, and pathway analysis of TCGA tumor datasets were performed using R packages. GEO data analysis was conducted for differential expression analysis and visualization. Gene Ontology (GO) and Kyoto Encyclopedia of Genes and Genomes (KEGG) analyses were performed using the Metascape database [26].

### Human RCC specimens

The specimens utilized in this study encompassed a pool of 40 cases of renal clear cell carcinoma tissues, carefully paired with corresponding tumor tissues and adjacent normal tissues. These samples were preserved within the tissue bank of the First Affiliated Hospital of Zhengzhou University. The subject cohort comprised individuals who underwent the diagnosis and management of primary renal cell carcinoma at The First Affiliated Hospital of Zhengzhou University during the period spanning from January 2020 to December 2020. (2022-KY-1089-001)

### Cell lines, cell culture, and transfection

786-O, ACHN, 769-P and HK-2 cell lines were obtained from the Cell Bank of the Chinese Academy of Sciences (Shanghai, China) and are described in detail in Supplementary Table 1. RPMI-1640 medium supplemented with 10% fetal bovine serum (FBS) was used to culture 786-O and 769-P cells, while MEM medium supplemented with 10% FBS was used for ACHN cells, and DMEM/F12 medium supplemented with 10% FBS was used for HK-2 cells. All of the cell lines were maintained within a temperature of 37 degrees Celsius, and within a 5% CO2 incubator. The cells were cultured for use in the logarithmic growth phase. Transfection was performed using Lipofectamine 2000 (Thermo Fisher Scientific Inc. Waltham, USA) as per the manufacturer's protocol.

### shRNA-mediated gene knockdown

Lentiviral vectors and the concentrated lentivirus were acquired from Vigene Biosciences, Inc. (Shandong, China). Lentiviral vectors comprising three shRNA constructs aiming at *GDF15* (sh#1-*GDF15*, sh#2-*GDF15*, and sh#3-*GDF15*, Supplementary table 2) were utilized to suppress *GDF15* expression. The transfection was conducted using jetPolygree transfection reagent (Polyplus-transfection, Illkirch, France), following the instructions provided by the manufacturer.

### Promoter luciferase assay

LIF luciferase reporter plasmids were obtained from OBiO Technology (Shanghai, China). For reporter constructs, the 860-bp promoter region (132/982) of the *GDF15* gene was cloned into the pGL4.1 firefly luciferase reporter vector. phRL-TK containing the Renilla luciferase gene was used as an internal standard for transfection efficiency. Cells were seeded into 24-well plates at a density of 1×10^5 cells/well and co-transfected with 500 ng luciferase construct plasmid or an empty reporter vector DNA and 0.05 ug phRL-TK using jetPEI transfection reagent for 48 hours (Polyplus-transfection). The cells were then lysed, and the luciferase activity was examined with the dual-luciferase reporter assay kit (Transgen Biotech, Beijing, China.For the dual-luciferase reporter assay, cells were co-transfected with the indicated reporter plasmid, *LMX1B* overexpression plasmid, and Renilla luciferase plasmid as an internal control. After 48 h of transfection, firefly and Renilla luciferase activities were measured using a dual-luciferase reporter assay system.) on a microplate reader with a multi-wavelength measurement system (Synergy LX, BioTek Instruments, Inc., Winooski, VT, USA). Luciferase activity was calculated relative to the Renilla luciferase activity.

### Colony formation assay

A total of 2×10^3 RCC cells were seeded across 6-well plates, with the intent to assess the proliferation capability of these cells. After 10 days, the RCC cells were subjected to fixation using a 1% crystal violet stain solution, and the number of colonies was counted.

### Reactive oxygen species (ROS) assay

The H2DCFDA (DCFH-DA, DCFH) ROS fluorescence probe (MKBio, Shanghai, China) was used to complement and verify intracellular ROS levels. Briefly, 2.5×10^5 cells were inoculated into a 6-well plate, then the medium was extracted, 10mM working solution and PBS were added, and the cells were cultured in a cell incubator for 40 minutes; the staining solution was removed and the cells were washed three times with cell culture medium. The cell culture medium was then added to the cells again and the level of ROS staining was assessed under a fluorescence microscope.

### Mitochondrial membrane potential assay

To assess mitochondrial membrane potential (MMP), we evaluated MMP using JC-1 dye acquired from Life Technologies Corporation (Carlsbad, CA). First, add 1 ml of JC-1 staining working solution, followed by 10 uM CCCP for positive control. Mix well and incubate at 37 C for 20 minutes in a cell incubator. After incubation, the supernatant was removed by suction and washed twice with JC-1 staining buffer (1X). Further, add 2 ml of cell culture medium and observe under a fluorescence microscope to determine the MMP.

### CCK-8 assay

Cells were seeded in 96-well plates at a density of 1×10^3 cells per well. At the designated time points (0, 1-, 2-, 3-, and 4- days post-transfection), 10 uL of CCK-8 reagent (Dojindo Molecular Technologies Inc., Kumamoto, Japan) was added to each well and incubated for 1.5 hours. The optical density of the wells was then measured at 450 nm (OD450) using a microplate reader.

### Cell migration and invasion assays

The migration and invasion abilities of cells were perceived using 8 um of polycarbonate membrane transwell plates (Corning Inc., Corning, NY, USA), with uncoated (for migration assay) and coated matrigel (for invasion assay). Briefly, 2×10^5 cells/mL cell suspensions were placed into the upper chambers with 200 uL of medium in each well. The lower chambers were filled with 600 uL of medium containing 20% FBS. After being incubated for 24 or 48 h at 37 C and 5 % CO2, the inner surface cells of the upper chamber were wiped with a cotton swab. The upper chambers were then fixed with 4 % paraformaldehyde and stained with 0.1 % crystal violet (Sigma, St.Louis, MO, USA), taking images under 200 x microscope.

### Apoptosis assay

The annexin V-FITC/PI apoptosis assay kit was purchased from Sharp (Beijing, China). Cells were collected and spun in a centrifuge tube at 1000-2000 rpm for 5 minutes. The supernatant was carefully removed and the cells were pre-cooled at 4 C with 1 ml PBS for resuspension. The binding buffer was diluted 1:4 with deionized water and the cells were resuspended with 250ul of the diluted binding buffer, adjusting the concentration to 1×10^6/ml. To perform the assay, 100 uL of the cell suspension was added to a 5 mL flow-through tube and gently mixed with 5 uL of Annexin V-FITC. The tube was then incubated for 10 minutes at room temperature in the dark. Next, 10 uL of propidium iodide solution was added and gently mixed in the reaction tube for 5 minutes. Finally, 400 uL of PBS was added to the reaction tube. The cells were resuspended and kept away from light. Flow cytometry was used for analysis.

### Quantitative real-time polymerase chain reaction

Extraction of total RNA using TRIzol reagent (Invitrogen). The cDNA was prepared with NovoScript Plus All-in-one 1st Strand cDNA Synthesis SuperMix (gDNA Purge) (Novoprotein, Shanghai, China). Gene transcripts were quantitated using the QuantStudio Three Real-Time PCR System (Thermo Fisher) and the NovoStart SYBR gPCR SuperMix Plus (Novoprotein), with *GAPDH* as an internal control. The relative mRNA expression level of the target gene was calculated using the comparative 2^-DeltaDeltaCt method. Briefly, the cycle threshold (Ct) value of the target gene was first normalized to the endogenous reference gene *GAPDH* (DeltaCt = Ct target - Ct reference). Subsequently, DeltaCt for each experimental sample was calibrated against the mean DeltaCt of the control group (DeltaDeltaCt = DeltaCt sample - mean DeltaCt control). The relative expression fold change was derived as 2^-DeltaDeltaCt. The primers sequence used during our quantitative real-time polymerase chain reaction (qRT-PCR) test is shown in Supplementary Table 3.

### Western blot

RIPA buffer (cat. no. R0010; Solarbio) was used to lyse cells, and protein concentrations were determined with a bicinchoninic acid protein assay kit (Beijing Leagene Biotech Co., Ltd.). Protein samples (25 ug/ lane) were separated by SDS-PAGE on 10% gels and then transferred onto PVDF membranes. The membranes were blocked by protein-free rapid block buffer (Epizyme Pharmaceutical Biotechnology Co., LTD. Cambridge, MA) for 30 minutes at room temperature and then incubated with primary antibodies overnight at 4 C. Following primary antibody incubation, the membranes were incubated with secondary antibody at room temperature for 1 h. Then membranes were scanned by an imaging system (ODYSSEY CLx, Gene Company limited), and densitometry was quantified using Image Studio Lite (LI-COR Biosciences). First, we calculate the gray values of the bands using ImageJ. Then, determine the relative protein levels by normalizing the intensity of the target protein band in each sample to the intensity of the internal control beta-Actin. Subsequently, compare the normalized values of the experimental group with the average normalized values of the control group to calculate the fold change. Information on the antibodies for which we did western blotting is recorded in Supplementary Table 1.

### ChIP-qPCR

ChIP-qPCR analysis was performed on 786-O cells, adhering to the instructions provided by the SimpleChIP Enzymatic Chromatin IP Kit (Magnet Beads) (9003, Cell Signaling Technology). We cross-linked 5×10^6 cells with 1% formaldehyde for 10 min at room temperature, halting the reaction with 2 ml of 10x glycine for 5 min. Cells were then washed with PBS, lysed in cold Buffer A supplemented with DTT and PIC, and incubated on ice for 10 min. Post-centrifugation, the nuclear pellets were digested with micrococcal nuclease in Buffer B containing DTT at 37 C for 20 min, with the reaction quenched by 50 mM EDTA. The nuclear fractions were then sonicated to fragment the nucleosomes, using a Xinzhi sonicator with a 1/8-inch probe for three 20-s intervals. A 2% aliquot of this sonicated material was set aside as an input control. Antibodies targeting *LMX1B* (ab180929, Abclone), and IgG isotype control (3900, Cell Signaling Technology) were then introduced to the sonicated chromatin and incubated overnight at 4 C. Upon addition of Protein G Magnetic Beads, the mixtures were incubated for an additional two hours at 4 C. Following magnetic separation and successive washes, DNA was eluted from the beads, and cross-links were reversed at 65 C in the presence of Proteinase K. The DNA was then purified using spin columns and quantified by qPCR to evaluate enrichment.

### In vivo tumor xenograft model

All SCID mice were purchased from Sipeifu Company (Beijing, China). Matrigel (Becton, Dickinson and Company, New Jersey, USA) was used to combine 1×10^6 cells in a 1:1 ratio. This mixture was then carefully injected subcutaneously into the mouse at a 45 ° angle using a 1ml syringe. After injection, the puncture site was gently compressed with the index finger for approximately one minute. The mice were then returned to their habitat. Sixty days after injection, the mice were humanely euthanized. The tumor masses were excised for photographic documentation. (2022-KY-1089-001)

### Statistical analysis

Computational and statistical analyses were conducted in R (v4.3.1). Associations between continuous variables were determined using Spearman rank correlation. Comparisons involving multiple groups were performed with the Kruskal-Wallis test, whereas pairwise differences were assessed using the Wilcoxon rank-sum test. Multiple-comparison adjustment was implemented according to the Benjamini-Hochberg procedure. For experimental data, differences between groups were discerned using Student's t-tests or ANOVA, unless otherwise specified. Data are presented as mean ± SD, derived from a minimum of three independent experiments. Analyses were conducted utilizing GraphPad Prism 7.0 (La Jolla, USA). Statistical significance was defined as a two-sided P value below 0.05.

## Results

### Single-cell transcriptomic profiling reveals *LMX1B* as a tumor suppressor in renal cell carcinoma

To dissect the cellular heterogeneity and spatial architecture of renal cell carcinoma (RCC) at an unprecedented resolution, we performed integrated single-cell RNA sequencing (scRNA-seq) and spatial transcriptomics (ST) analyses on RCC specimens. By analyzing scRNA-seq data from three independent RCC cohorts (GSE230358, GSE230359, and GSE230360), we identified eight major cell populations, including B/plasma cells, endothelial cells, epithelial cells, fibroblasts, mast cells, myeloid cells, proliferating cells, and T/NK cells, through unsupervised clustering and marker gene annotation (Fig. 1A-C). The tracks plot and density visualizations of canonical lineage markers, including *KRT8* and *KRT18* for epithelial cells, *CD79A* for B/plasma cells, *ACTA2* for fibroblasts, *CD68* for myeloid cells, *TPSAB1* for mast cells, *MKI67* for proliferating cells, *CD3D* for T/NK cells, and *PECAM1* for endothelial cells, confirmed the fidelity of our cell type assignments (Fig. 1D, E). Comparative analysis of cell composition across datasets and tissue origins revealed substantial alterations in the tumor microenvironment (TME) architecture between normal adjacent kidney tissue and tumor tissue (Fig. 1F), underscoring the alterations in cellular composition were observed during RCC progression.

**Fig. 1:**
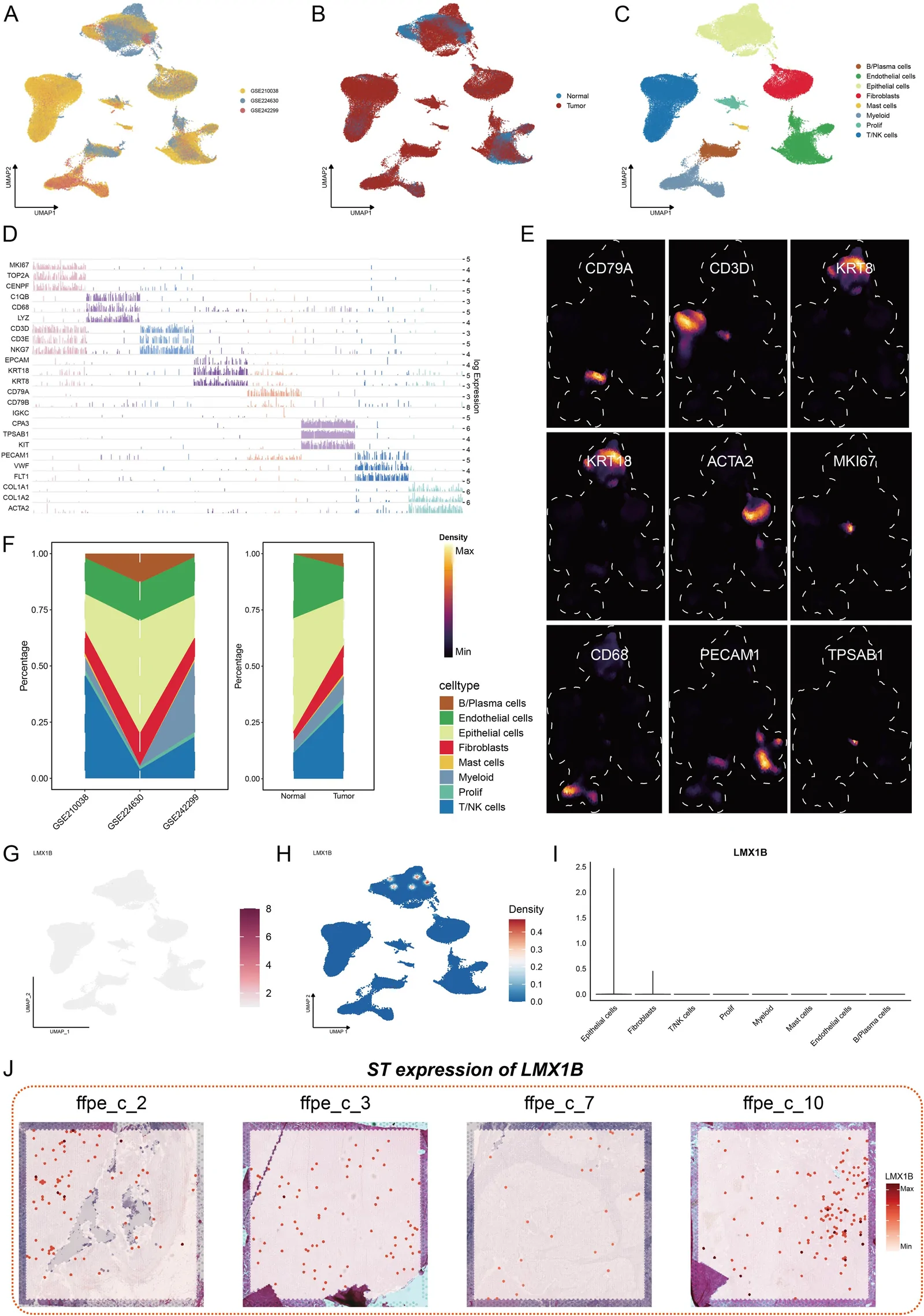
**Single-cell and spatial transcriptomic profiling of renal cell carcinoma. (A)** UMAP visualization of single-cell RNA sequencing data from three renal cell carcinoma (RCC) samples (GSE230358, GSE230359, and GSE230360), colored by sample origin. **(B)** UMAP visualization showing the distribution of cells from normal adjacent kidney tissue (blue) and tumor tissue (red). **(C)** UMAP visualization of all cells annotated by cell type, including B/Plasma cells, Endothelial cells, Epithelial cells, Fibroblasts, Mast cells, Myeloid cells, Prolif (proliferating cells), and T/NK cells. **(D)** Tracks plot showing cell type-specific markers (rows) across identified cell types (columns), grouped by cell type (e.g., B/Plasma cells, T/NK cells). 100 randomly selected cells from each cell type were used for plotting. Scale on the y-axis (right), normalized, log-transformed transcript counts in each cell. **(E)** Density plots showing expression distributions of representative marker genes across major cell types, including epithelial cells (*KRT8, KRT18*), B/plasma cells (*CD79A*), fibroblasts (*ACTA2*), myeloid cells (*CD68*), mast cells (*TPSAB1*), proliferating cells (*MKI67*), T/NK cells (*CD3D*), and endothelial cells (*PECAM1*). **(F)** Bar plots illustrating the compositional distribution of major cell populations across distinct single-cell RNA-sequencing datasets (left) and tissue origins (right). **(G)** Feature plot showing *LMX1B* expression on the single-cell UMAP. **(H)** Density plot showing the distribution of *LMX1B* expression on the single-cell UMAP. **(I)** Violin plot showing the expression levels of *LMX1B* across different cell types identified by single-cell analysis. **(J)** Spatial expression maps of *LMX1B* in four representative ST samples (ffpe_c_2, ffpe_c_3, ffpe_c_7, and ffpe_c_10), with color intensity indicating expression levels from minimum (light) to maximum (dark red/purple).

Notably, feature plot and density analyses of *LMX1B* expression indicated that this transcription factor was primarily detected in epithelial cell clusters, with relatively higher expression levels observed in normal epithelial cells compared with tumor-derived epithelial cells. However, the expression pattern exhibited certain heterogeneity among epithelial populations, which may reflect the intrinsic diversity of epithelial cell states and differences in transcriptomic visualization. (Fig. 1G, H). Violin plot quantification further demonstrated that *LMX1B* expression was highest in epithelial cells and fibroblasts, with significantly diminished levels in tumor-associated epithelial populations (Fig. 1I). To complement the single-cell findings, spatial transcriptomic mapping of *LMX1B* across four representative formalin-fixed paraffin-embedded (FFPE) samples (ffpe_c_2, ffpe_c_3, ffpe_c_7, and ffpe_c_10) revealed distinct spatial expression patterns, with *LMX1B* signals enriched in regions annotated as epithelial compartments based on their transcriptional characteristics and established epithelial marker expression (Fig. 1J). These findings suggest that *LMX1B* expression is preferentially associated with epithelial regions in RCC tissues.These integrative single-cell and spatial analyses establish *LMX1B* as a candidate tumor suppressor whose expression is spatially restricted and downregulated during RCC pathogenesis.

### *LMX1B* is downregulated in RCC tissues and correlates with malignant progression

To validate the bioinformatic findings, we performed qRT-PCR analysis on 40 paired RCC and adjacent normal tissue samples. Consistent with the scRNA-seq and ST data, *LMX1B* was markedly downregulated in RCC tissues compared to matched normal tissues (Fig. 2A). This observation was independently corroborated by TCGA data analysis, which revealed significantly diminished *LMX1B* expression in primary RCC tumors relative to normal kidney tissues (Fig. 2B). Importantly, *LMX1B* expression exhibited a substantial inverse correlation with tumor grade and lymphatic metastasis status, with progressively lower expression observed in higher-grade tumors (Grade 1–4) and node-positive (N1) cases compared to normal tissues and node-negative (N0) tumors, respectively (Fig. 2C, D). Analysis of the Cancer Cell Line Encyclopedia (CCLE) database further demonstrated that *LMX1B* mRNA levels were notably reduced in the 786-O and ACHN RCC cell lines (Fig. 2E). Both qRT-PCR and western blot analyses confirmed the subdued expression of *LMX1B* in RCC cell lines (786-O, ACHN, and 769-P) relative to the normal renal tubular epithelial cell line HK-2 (Fig. 2F, G). Collectively, these multi-platform analyses accentuate the tumor-suppressive potential of *LMX1B* in RCC and its adverse association with malignant progression.

**Fig. 2:**
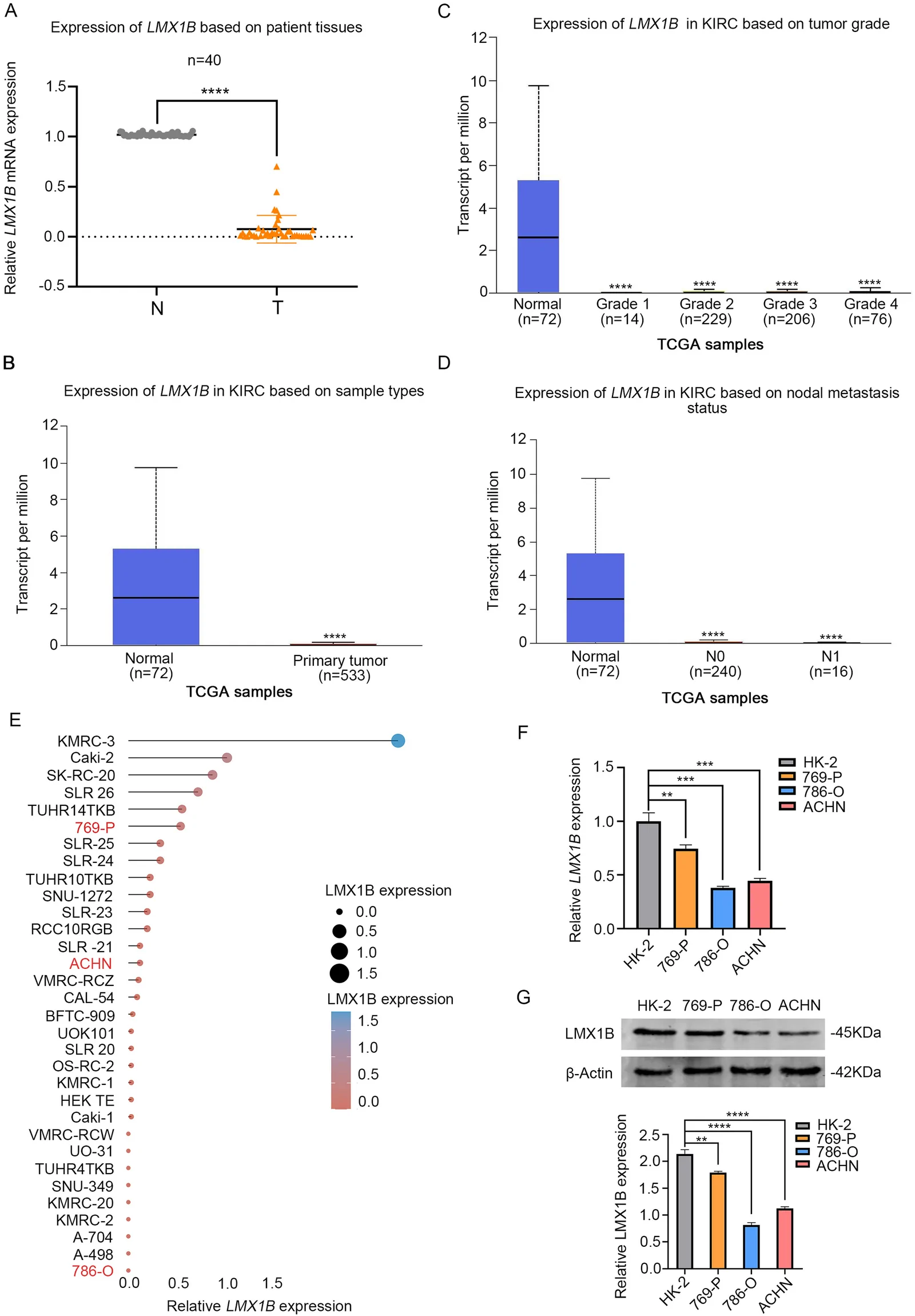
***LMX1B* downregulated in RCC tissues. (A)** Comparative *LMX1B* expression across 10 matched RCC and adjacent nontumor tissues. **(B-D)** Analysis of *LMX1B* expression using data from TCGA. **(E)** Expression profiling of *LMX1B* mRNA derived from the CCLE. **(F)** qRT–PCR illustrating relative *LMX1B* expression across different cell lines. **(G)** Western blot representation of *LMX1B* protein levels in various cell lines.

### Cell-cell communication and spatial deconvolution reveal *LMX1B*-associated epithelial cells as central hubs in the RCC microenvironment

To elucidate the intercellular communication networks orchestrated by *LMX1B*-expressing epithelial cells within the RCC TME, we employed CellChat-based ligand-receptor interaction analysis. Circle plots depicting interaction number and interaction weight revealed that *LMX1B*-associated epithelial cells (*LMX1B*-Epi) served as prominent signaling hubs, engaging in extensive bidirectional communication with fibroblasts, endothelial cells, myeloid cells, and T/NK cells (Fig. S1A). Dot plot visualization of significant ligand-receptor pairs emanating from *LMX1B*-Epi to other cell types identified multiple critical signaling axes, including *VEGFA*-*VEGFR1/VEGFR2*-mediated angiogenic signaling, *SPP1*-*CD44/ITGAV+ITGB1*-mediated adhesive interactions, and *GDF15*-*TGFBR2*-mediated growth regulatory pathways (Fig. S1B). Conversely, incoming signals to *LMX1B*-Epi were enriched for *TNF*-*TNFRSF1A*-mediated inflammatory signaling, *SPP1*-(*ITGAV+ITGB1*)-mediated matrix interactions, and *PTN*-*SDC4*/*NCL*-mediated growth factor signaling (Fig. S1C), indicating that *LMX1B*-Epi cells are both active signal emitters and receivers within the TME.

To spatially contextualize these communication networks, we performed RCTD (Robust Cell Type Decomposition) deconvolution on spatial transcriptomics data from the ffpe_c_2 sample. Hematoxylin and eosin (H&E) staining and Seurat cluster mapping provided the histological and transcriptional framework for spatial analysis (Fig. S1D, E). RCTD deconvolution revealed the spatial distribution and proportional abundance of each cell type across the tissue section, demonstrating that *LMX1B*-negative epithelial cells (*LMX1B*.neg.Epi) occupied distinct spatial niches compared to B/plasma cells, endothelial cells, fibroblasts, mast cells, myeloid cells, proliferating cells, and T_NK cells (Fig. S1F). To further investigate the spatial colocalization relationships between cell types, we conducted mistyR spatial colocalization analysis across three spatial contexts: intra-spot (global), juxta-5 (5-spot radius), and para-15 (15-spot radius). The global spatial correlation heatmap and network plot revealed significant spatial associations between *LMX1B*.neg.Epi cells and fibroblasts, as well as between myeloid cells and T_NK cells. These spatial relationships suggest that *LMX1B*.neg.Epi cells are preferentially located within fibroblast-enriched microenvironmental niches, indicating potential epithelial–stromal interactions in RCC tissues. In addition, the observed proximity between myeloid and T_NK cells reflects the coordinated organization of immune cell compartments within the tumor microenvironment. (Fig. S1G). At the juxta-5 scale, *LMX1B*.neg.Epi exhibited strong spatial proximity to myeloid cells and fibroblasts (Fig. S1H), while the para-15 analysis demonstrated broader spatial associations involving *LMX1B*.neg.Epi, fibroblasts, and T_NK cells (Fig. S1I). These spatially resolved analyses demonstrate that *LMX1B*-associated epithelial cells occupy specialized niches within the RCC TME and engage in spatially organized intercellular communication networks that may govern tumor progression.

### HdWGCNA identifies co-expression modules and functional pathways associated with *LMX1B* in RCC epithelial cells

To investigate the transcriptional programs regulated by *LMX1B* in RCC epithelial cells, we performed hierarchical differential weighted gene co-expression network analysis (hdWGCNA) on *LMX1B*-associated epithelial cell populations. Scale-free topology and connectivity analyses identified a soft-thresholding power of 7 as the optimal parameter for network construction (Fig. S2A). Hierarchical clustering of the resulting gene co-expression network revealed five distinct modules (M1–M5), each characterized by unique gene membership profiles (Fig. S2B). Module eigengene analysis identified the top hub genes with highest module membership (kME) for each module: M1 was enriched for stress-response genes including *JUND, HSP90AA1*, and *FOSB*; M2 contained metabolic regulators such as *LGALS2, NAT8*, and *CYB5A*; M3 comprised acute-phase response genes including *CP, APCS*, and *SERPINF2*; M4 was dominated by ribosomal proteins (*RPL41, RPL31, RPL7*); and M5 featured mitochondrial ATP synthesis genes including *ATP5MC2, ATP5F1E*, and *ATP5F1D* (Fig. S2C).

UMAP visualization of module eigengene scores across epithelial cells revealed distinct spatial distribution patterns for each module, with M1 and M2 broadly distributed, while M3, M4, and M5 exhibited more restricted expression domains (Fig. S2D). Network visualization of module relationships between normal and tumor *LMX1B*-associated epithelial cells demonstrated that M1 (stress response) and M2 (metabolism) were the largest modules and exhibited strong inter-module connectivity, whereas M5 (mitochondrial function) formed a distinct cluster (Fig. S2E). UMAP overlays of average module expression and percent of expressed genes further confirmed the differential module activation between normal and tumor epithelial populations (Fig. S2F). Gene Ontology (GO) biological process enrichment analysis revealed that M1 was significantly enriched in nucleotide-binding oligomerization domain (NOD)-like receptor signaling and stress-induced intrinsic apoptotic signaling; M2 in cellular respiration and mitochondrial ATP synthesis coupled electron transport; M3 in response to unfolded protein; M4 in cytoplasmic translation; and M5 in proton motive force-driven ATP synthesis and ATP biosynthetic process (Fig. S2G). Strikingly, violin plot comparisons of module eigengene scores between normal and tumor *LMX1B*-associated epithelial cells revealed significant differential activation of all five modules (****P < 0.0001), with M1, M3, M4, and M5 elevated in tumor cells, while M2 showed distinct expression patterns (Fig. S2H). These hdWGCNA findings illuminate the modular transcriptional architecture governed by *LMX1B* in RCC epithelial cells and highlight mitochondrial dysfunction and metabolic reprogramming as central features of *LMX1B*-associated tumor biology.

### *LMX1B* inhibits proliferation and invasion of RCC cells *in vitro*

To functionally validate the tumor-suppressive role of *LMX1B* identified through our multi-omics analyses, we established *LMX1B*-overexpressing (*LMX1B* OE) 786-O and ACHN cell lines. qRT-PCR and western blot analyses confirmed robust *LMX1B* overexpression at both mRNA and protein levels (Fig. 3A, B). Time-course western blot analysis revealed that *LMX1B* protein expression increased progressively following transfection, plateauing after 72 hours (Fig. S3A). Transwell migration and invasion assays demonstrated that *LMX1B* overexpression significantly suppressed the migratory and invasive capabilities of both 786-O and ACHN cells (Fig. 3C). Proliferation assays, including CCK-8, colony formation, and EDU incorporation, consistently revealed that *LMX1B* overexpression markedly inhibited RCC cell proliferation (Fig. 3D-F; Fig. S3B). These *in vitro* findings corroborate the bioinformatic predictions and establish *LMX1B* as a functional suppressor of RCC malignant phenotypes.

**Fig. 3:**
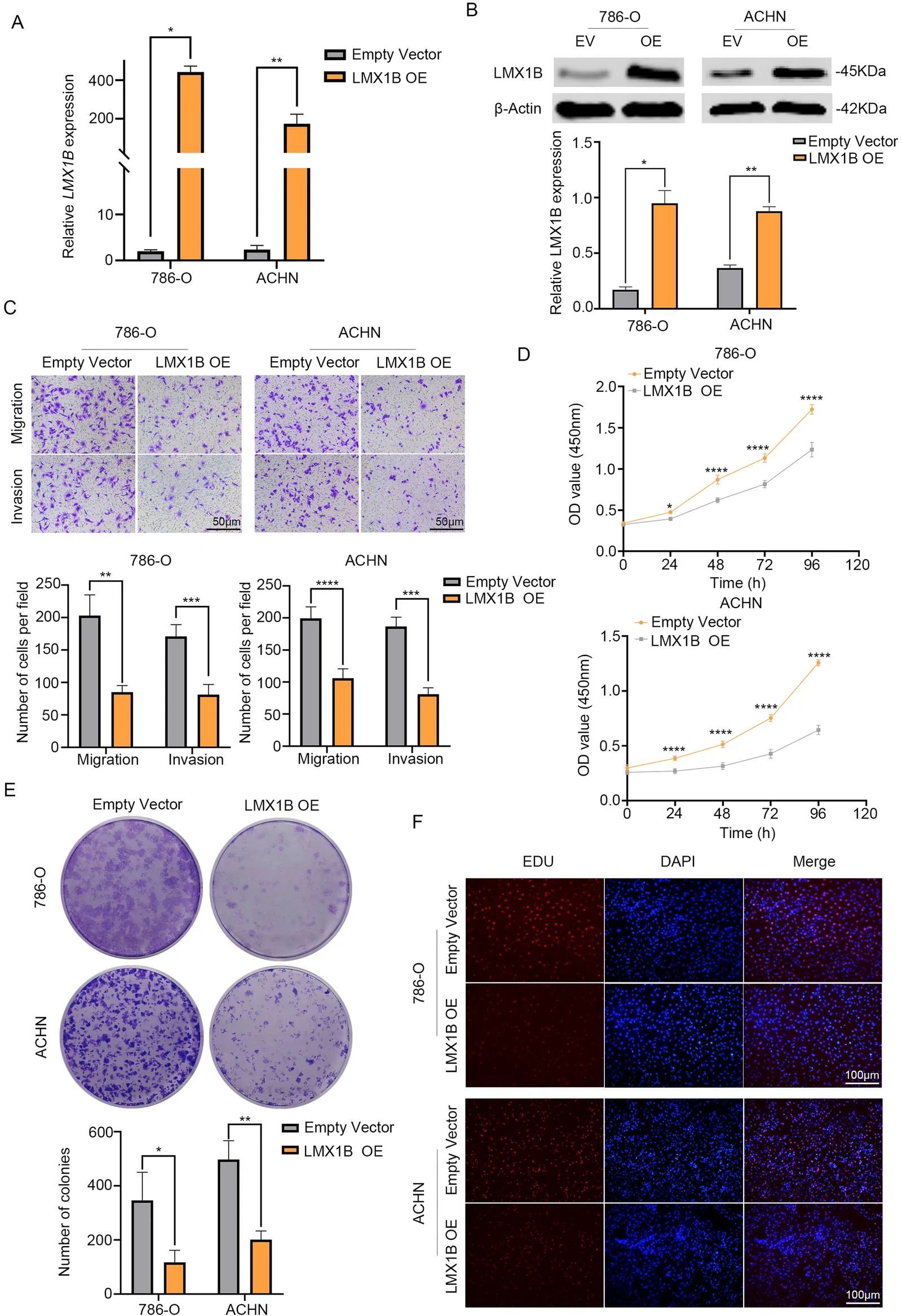
***LMX1B* inhibited proliferation and invasion of RCC cells *in vitro*. (A, B)** Validation of *LMX1B* overexpression using both qRT-PCR and western blot analyses. **(C)** Transwell assays elucidating the impact of *LMX1B* on the migratory and invasive capabilities of RCC cells. **(D-F)** Functional assays including CCK-8, colony formation and EDU were used to evaluate the proliferative potential of *LMX1B* overexpression in RCC cells. All experiments were performed with three independent biological replicates (n = 3), and data are presented as mean ± SEM.

### *LMX1B* transcriptionally activates *GDF15* by directly binding to its promoter

To elucidate the molecular mechanism underlying *LMX1B*-mediated tumor suppression, we integrated GEO dataset (GSE12008) analysis, TCGA differential expression screening, and JASPAR transcription factor binding prediction to identify downstream targets of *LMX1B* (Fig. 4A). Differential gene expression analysis of the GSE12008 dataset identified 43 significantly upregulated genes (logFC ≥ 2, P ≤ 0.05) in *LMX1B*-overexpressing cells (Fig. 4B, C). Intersection with TCGA data revealed that only *GDF15* and *SUCNR1* among these 43 DEGs exhibited low expression in RCC tumor tissues (Fig. 4D). Given that *SUCNR1* has been reported to promote cancer progression, we selected *GDF15* for further mechanistic investigation.

**Fig. 4:**
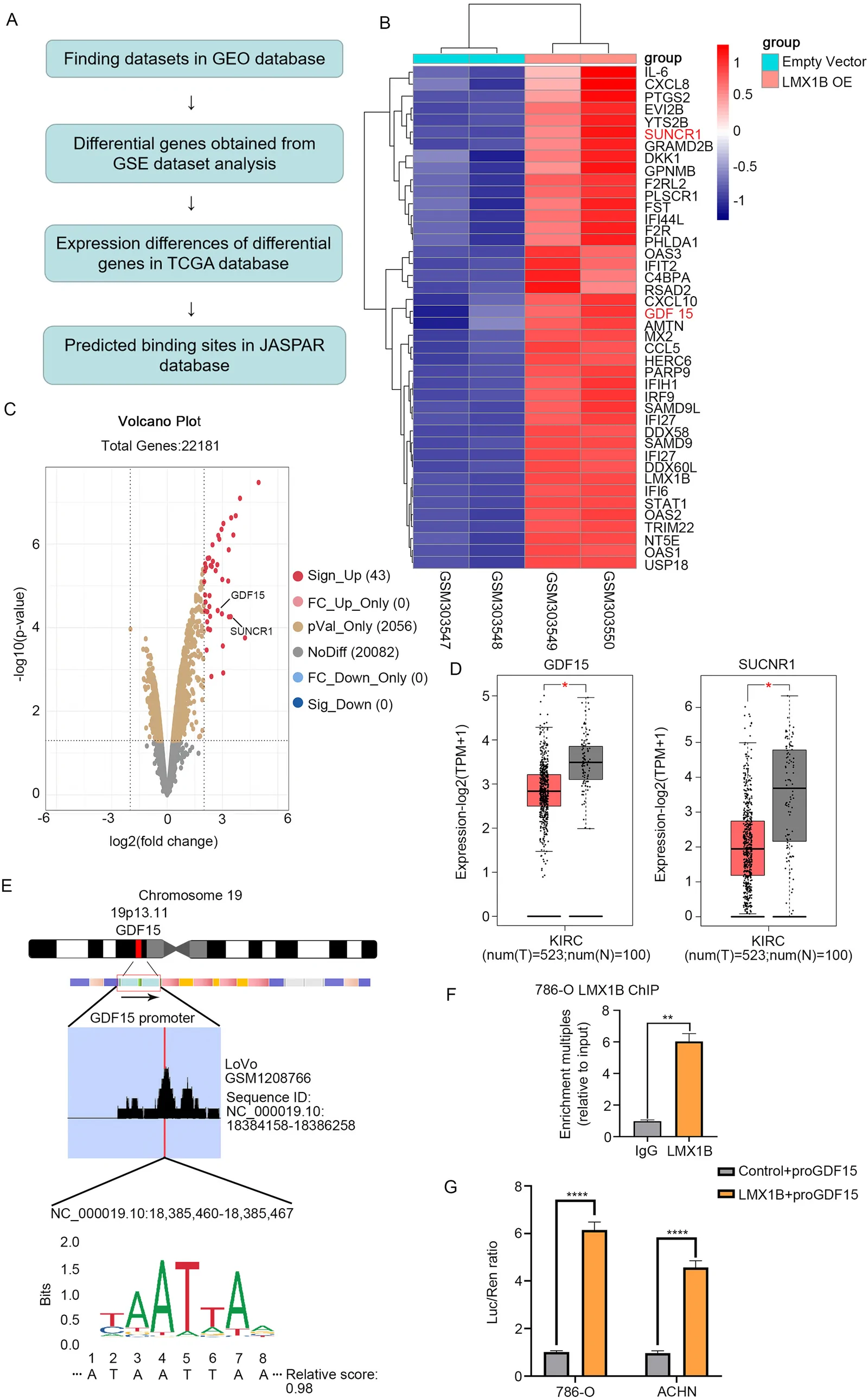
***LMX1B* regulated the transaction of *GDF15*. (A)** Schematic representation of the approach used to identify downstream targets of *LMX1B*. **(B, C)** Differential gene expression analysis of the GSE12008 dataset visualized using a heatmap and scatterplot, respectively, processed with R(n=10 samples). **(D)** Comparative analysis of *GDF15* and *SUCNR1* expression in RCC within TCGA database(n = 546 patients). **(E)** Illustration of the chromosomal positioning of the *GDF15* promoter and the result of the ChIP analysis of the *LMX1B* gene. Predicted *LMX1B* binding sites on the *GDF15* promoter as determined by the JASPAR database. **(F)***LMX1B* specifically binds to the target genomic region as validated by ChIP-qPCR(n = 3). **(G)***LMX1B* directly targets and binds to *GDF15* promoter as demonstrated by Dual-Luciferase Assays(n = 3).

Analysis of the NCBI database and JASPAR prediction identified a putative *LMX1B* binding motif (AATATAAA, relative score: 0.98) within the *GDF15* promoter region on chromosome 19 (19p13.11) (Fig. 4E). ChIP-qPCR analysis using a specific *LMX1B* antibody demonstrated significantly higher enrichment at the *GDF15* promoter region compared to the IgG negative control in 786-O cells (Fig. 4F). Furthermore, dual-luciferase reporter assays in 786-O and ACHN cells co-transfected with the *LMX1B* expression vector and *GDF15* promoter constructs revealed that *LMX1B* significantly enhanced *GDF15* promoter-driven luciferase activity (Fig. 4G). qRT-PCR analysis confirmed that *LMX1B* overexpression upregulated *GDF15* mRNA expression (Fig. S3C). Collectively, these findings demonstrate that *LMX1B* directly binds to the *GDF15* promoter and transcriptionally activates its expression in RCC cells.

### *GDF15* inhibits proliferation and invasion of RCC cells *in vitro* and *in vivo*

To investigate the functional consequences of *LMX1B*-mediated *GDF15* activation, we performed loss-of-function and gain-of-function studies. qRT-PCR and western blot analyses validated the efficacy of *GDF15* knockdown (sh-*GDF15*#1, sh-*GDF15*#2, sh-*GDF15*#3) and overexpression in 786-O and ACHN cells (Fig. 5A, B; Fig. S4A, B). Based on qRT-PCR efficiency, sh-*GDF15*#1 and sh-*GDF15*#2 were selected for subsequent experiments. Transwell assays demonstrated that *GDF15* knockdown significantly enhanced migration and invasion, whereas *GDF15* overexpression suppressed these malignant behaviors (Fig. 5C; Fig. S4C). CCK-8, colony formation, and EDU assays revealed that *GDF15* knockdown promoted proliferation, while *GDF15* overexpression inhibited RCC cell proliferation (Fig. 5D, E; Fig. S4D, E; Fig. S5A,B).

**Fig. 5:**
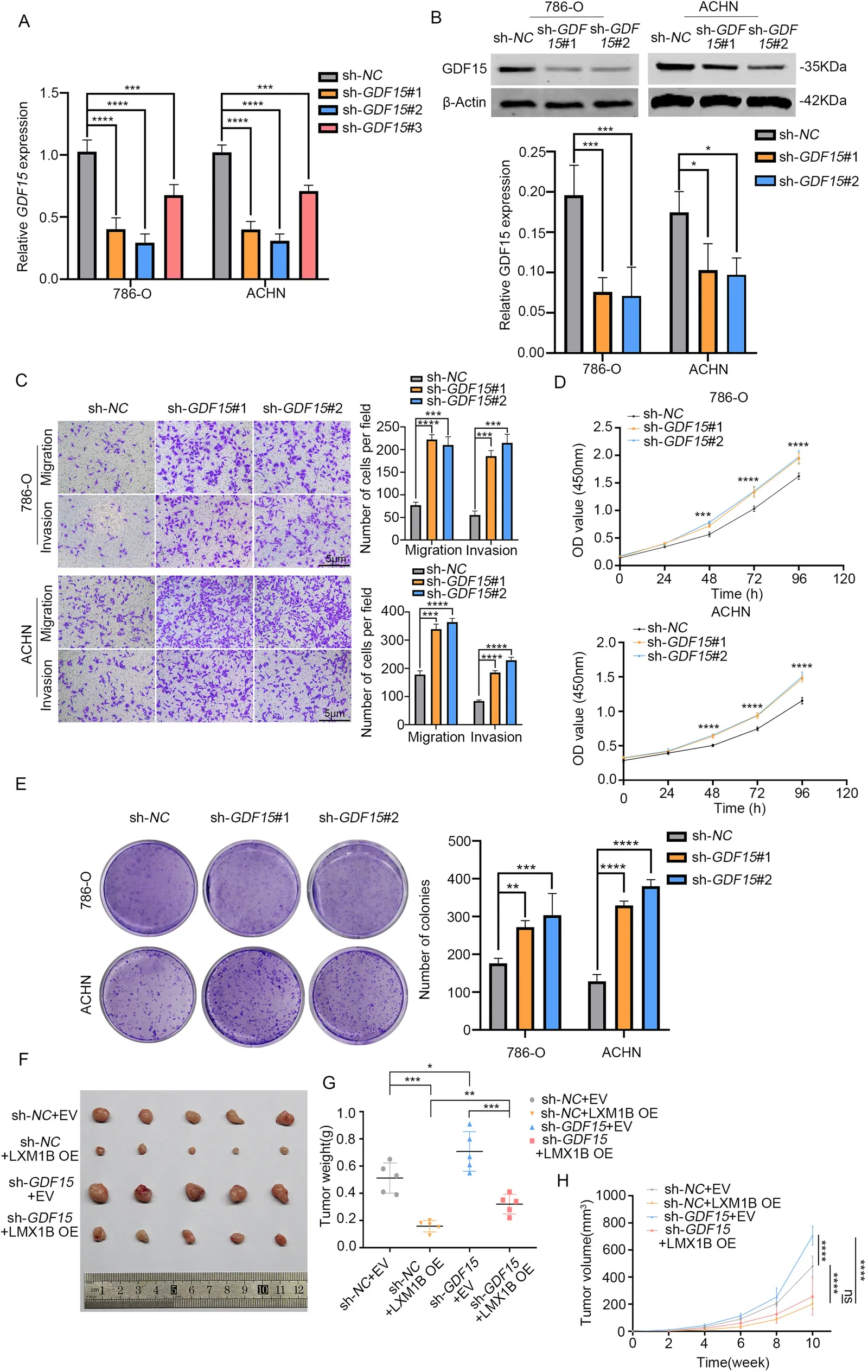
***GDF15* inhibited proliferation and invasion of RCC cells *in vitro*. (A)** qRT-PCR analysis demonstrates the efficiency of *GDF15* knockdown.Data are mean ± SEM(n = 3). **(B)** Western blotting shows *GDF15* expression levels after knockdown.Data are mean ± SEM(n = 3). **(C)** Transwell assays highlight the influence of *GDF15* on the migratory and invasive capabilities of RCC cells.Data are mean ± SEM(n = 3). **(D, E)** Proliferative potential of RCC cells following *GDF15* knockdown as assessed by CCK-8 and colony formation assays.Data are mean ± SEM(n = 3). **(F)** Visual representation of tumor growth in different experimental groups.Data are mean ± SEM(n = 5). **(G)** Graphical representation of xenograft tumor volumes (mm3) developed in SCID mice in different groups.Data are mean ± SEM(n = 5). **(H)** Average tumor weight at study endpoint (expressed in grams).Data are mean ± SEM(n = 5).

To assess the *in vivo* relevance of these findings, we performed subcutaneous xenograft experiments in SCID mice. *GDF15* knockdown significantly increased tumor growth, volume, and weight compared to the sh-NC control group (Fig. 5F-H). Importantly, concurrent overexpression of *LMX1B* reversed the enhanced tumorigenicity induced by *GDF15* knockdown, resulting in smaller tumors that were comparable to the control group (Fig. 5F-H). These *in vitro* and *in vivo* findings establish *GDF15* as a downstream effector of *LMX1B* that suppresses RCC progression.

### *GDF15* modulates mitochondrial apoptosis in human RCC cells

Given the hdWGCNA-identified enrichment of mitochondrial and apoptotic pathways in *LMX1B*-associated modules, we investigated whether *GDF15* regulates mitochondrial-mediated apoptosis. Flow cytometry analysis using Annexin V-FITC/PI staining revealed that *GDF15* knockdown significantly reduced the apoptotic cell population (Quadrants III and IV) in both 786-O and ACHN cells (Fig. 6A). JC-1 mitochondrial membrane potential (MMP) assays demonstrated that *GDF15* silencing led to increased MMP, as evidenced by enhanced red fluorescence and diminished green fluorescence, indicating reduced mitochondrial depolarization (Fig. 6B, C). Conversely, ROS fluorescence probe assays showed that *GDF15* knockdown significantly decreased intracellular ROS levels (Fig. 6D; Fig. S6A).

**Fig. 6:**
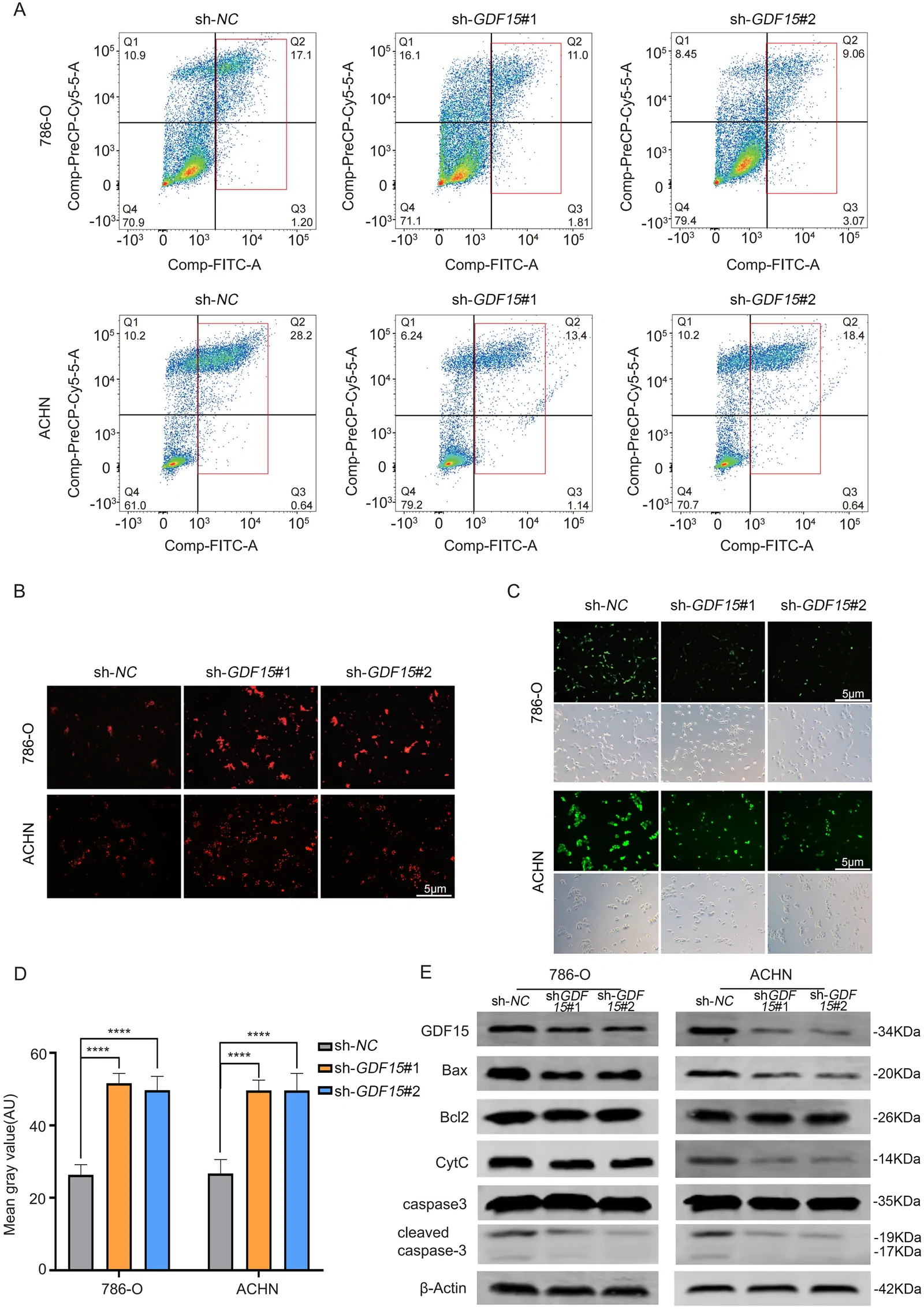
***GDF15* modulates mitochondrial apoptosis in human RCC cells. (A)** Flow cytometry analysis showing the apoptotic cell population in RCC samples. The x-axis represents Annexin V staining and the y-axis represents PI staining, with each quadrant delineating different cell populations: live, early apoptotic, late apoptotic and necrotic.Data are mean ± SEM(n = 3). **(B, C)** The JC-1 assay elucidates alterations in MMP. Viable cells, characterized by elevated MMP, emit red fluorescence, while apoptotic cells with diminished MMP radiate green fluorescence.Data are mean ± SEM(n = 3). **(D)** ROS assay showing the downregulation of reactive oxygen species (ROS) levels after *GDF15* knockdown. A decrease in fluorescence intensity indicates reduced ROS levels.Data are mean ± SEM(n = 3). **(E)** Western blot analysis showing the expression profiles of critical proteins associated with the apoptotic pathway, including Bax, Bcl-2, Cyt C and cleaved caspase-3, after *GDF15* knockdown.Data are mean ± SEM(n = 3).

Western blot analysis of apoptosis-related proteins revealed that *GDF15* knockdown suppressed the expression of pro-apoptotic Bax, cytochrome c (Cyt C), and cleaved caspase-3, while concurrently reducing the Bax/Bcl-2 ratio (Fig. 6E; Fig. S6B, C). Immunohistochemical staining of xenograft tumor tissues further confirmed that cleaved caspase-3 levels were diminished following *GDF15* knockdown (Fig. S6D, E). These findings collectively demonstrate that *GDF15* promotes mitochondrial apoptosis in RCC cells through ROS-mediated MMP depolarization and activation of the Bax/Bcl-2-caspase-3 cascade.

### *GDF15* positively regulates *ATP4B* expression in RCC cells

To identify downstream mediators of *GDF15*-induced tumor suppression, we stratified TCGA RCC data into high and low *GDF15* expression cohorts and performed differential gene analysis (logFC ≥ 1, P ≤ 0.05). The resulting volcano plot revealed *ATP4B* as one of the most significantly upregulated genes in the high-*GDF15* group (Fig. 7A). GO enrichment analysis of differentially expressed genes identified functional categories related to sodium ion transport, pH regulation, acute-phase response, and phosphagen metabolic processes (Fig. 7B; Table S4). Notably, *ATP4B* appeared recurrently within the top 20 enriched KEGG pathways analyzed via Metascape.

**Fig. 7:**
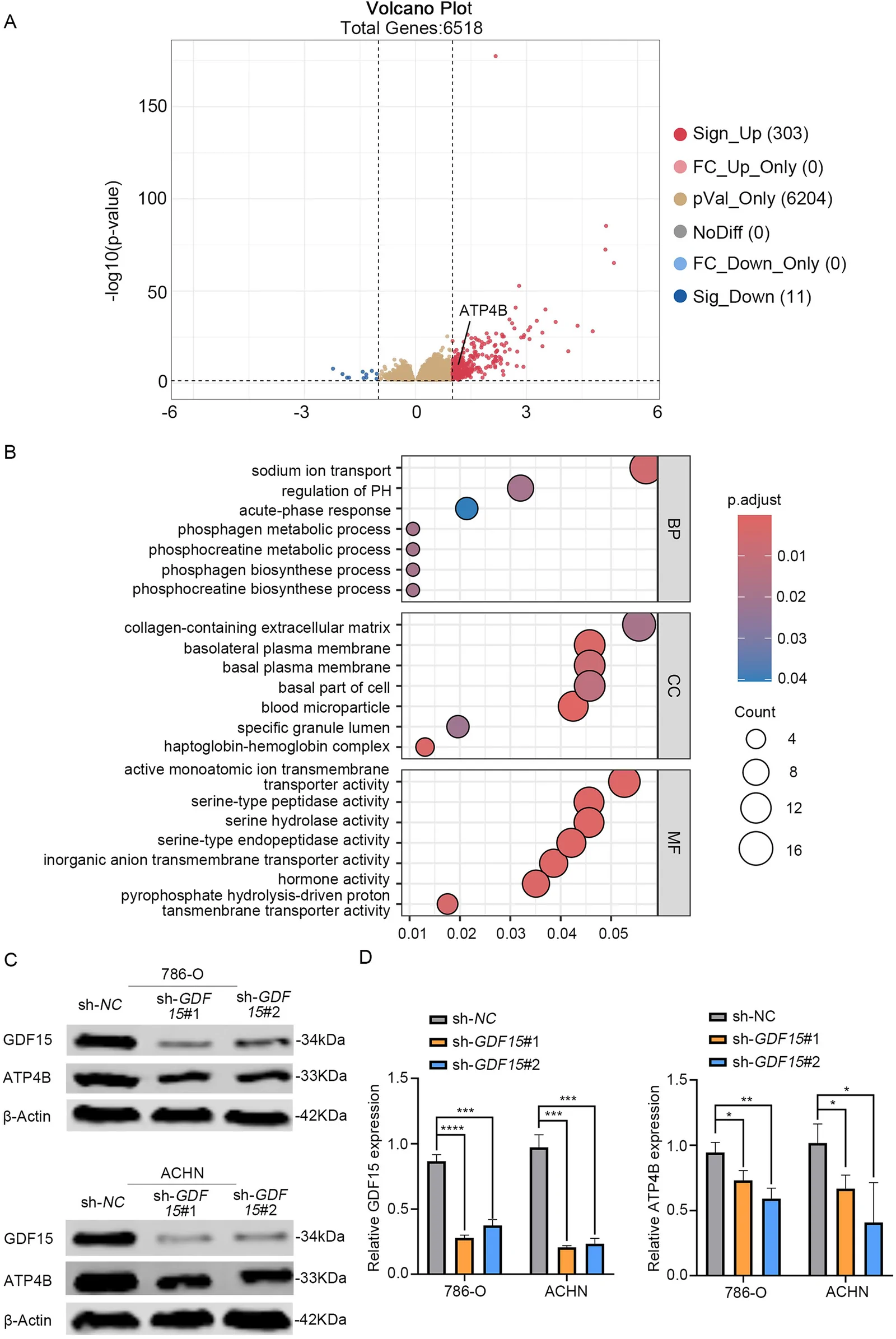
***GDF15* could regulate *ATP4B* in human RCC cells. (A)** Volcano plot visualized differential gene expression correlated with *GDF15* (log FC≥1), P<0.05.(n=324 samples). **(B)** Gene Ontology (GO) analysis showing the top functional categories enriched among the differentially expressed genes. **(C)** Representative western blot bands showing protein expression levels of *GDF15* and *ATP4B* after *GDF15* knockdown in RCC cells.Data are mean ± SEM(n = 3). **(D)** Quantitative analysis of Western blot results showing a significant decrease in *GDF15* and *ATP4B* protein levels after *GDF15* knockdown.Data are mean ± SEM(n = 3).

To validate this bioinformatic prediction, we performed western blot analysis following *GDF15* knockdown. The results demonstrated that *GDF15* silencing led to a concomitant decrease in *ATP4B* protein expression in both 786-O and ACHN cells (Fig. 7C, D). These findings robustly support the regulatory influence of *GDF15* on *ATP4B* expression in RCC.

### *GDF15* exerts tumor-suppressive effects through *ATP4B*-dependent mechanisms

To elucidate the functional relationship between *GDF15* and *ATP4B* in RCC progression, we conducted rescue experiments. qRT-PCR and western blot analyses confirmed that *ATP4B* overexpression restored *ATP4B* expression in *GDF15*-knockdown cells without affecting *GDF15* levels (Fig. 8A, B; Fig. S6F,G). Analysis and visualization of The Cancer Genome Atlas (TCGA) data using the UALCAN database, together with qRT‑PCR results from the 40 clinical RCC samples collected in this study, demonstrated that *ATP4B* expression was downregulated in RCC tissues, consistent with the expression pattern of *GDF15* (Fig. S6H).Transwell migration and invasion assays revealed that *ATP4B* overexpression significantly reversed the enhanced migratory and invasive capacities induced by *GDF15* knockdown in both 786-O and ACHN cells (Fig. 8C, D). CCK-8 and colony formation assays demonstrated that *ATP4B* overexpression rescued the proliferative inhibition caused by *GDF15* silencing (Fig. 8E-G).

**Fig. 8:**
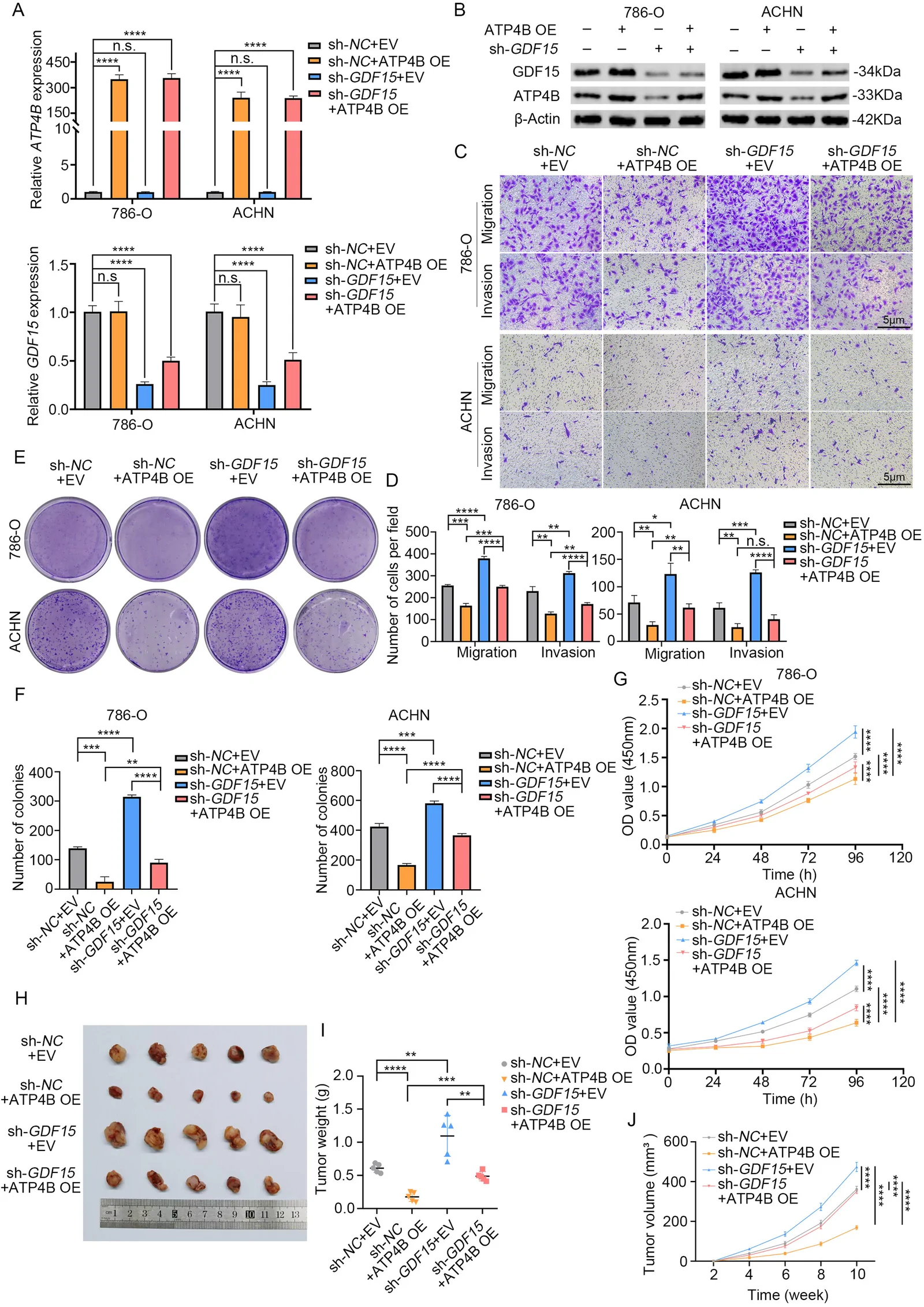
***GDF15* accelerates RCC cell suppressor effects by regulating *ATP4B in vitro*. (A)** The mRNA expression efficiency of *GDF15* and *ATP4B* in RCC cells was demonstrated through qRT-PCR results.Data are mean ± SEM(n = 3). **(B)** The protein expression efficiency of *GDF15* and *ATP4B* was shown in Western blot analyses.Data are mean ± SEM(n = 3). **(C, D)** The Transwell assays demonstrate the invasive and migratory capabilities of RCC cells under various conditions.Data are mean ± SEM(n = 3). **(E-G)** Proliferation capability of RCC cells was evaluated post modulation of *GDF15* and *ATP4B* through colony formation and CCK-8 assays. A graphical depiction displays variations in cell proliferation rates between the experimental conditions.Data are mean ± SEM(n = 3). **(H)** Representative images of subcutaneous tumors formed in SCID mice throughout all experimental groups demonstrate differences in tumor growth and size.Data are mean ± SEM(n=5). **(I)** This plot illustrates the volume of xenograft tumors (mm3) formed in SCID mice across different experimental groups.Data are mean ± SEM(n=5). **(J)** The bar graph presents the average final weight of tumors from each experimental group, measured in grams.Data are mean ± SEM(n=5).

In subcutaneous xenograft models, *GDF15* knockdown resulted in significantly increased tumor growth, volume, and weight compared to the sh-NC+EV control group. Strikingly, *ATP4B* overexpression completely reversed these oncogenic effects, restoring tumor parameters to levels comparable to or lower than the control group (Fig. 8H-J). These rescue experiments confirm that *GDF15* suppresses RCC progression through upregulation of *ATP4B*, establishing the *LMX1B*-*GDF15*-*ATP4B* axis as a critical regulatory cascade in RCC pathogenesis.

## Discussion

The tumor microenvironment of renal cell carcinoma represents a complex ecosystem in which malignant epithelial cells engage in dynamic, bidirectional communication with stromal, immune, and vascular compartments to foster tumor progression and therapeutic resistance. In this study, we leveraged the complementary power of single-cell RNA sequencing and spatial transcriptomics to construct a spatially resolved atlas of RCC cellular heterogeneity, revealing that the LIM homeodomain transcription factor *LMX1B* operates as a critical tumor suppressor whose downregulation dismantles epithelial-intrinsic transcriptional programs and disrupts the spatial organization of intercellular communication networks within the TME. Our multi-omic and functional analyses collectively establish the *LMX1B*–*GDF15*–*ATP4B* axis as a previously unrecognized regulatory cascade that governs mitochondrial homeostasis and apoptotic commitment in renal malignancy, with profound implications for prognostic stratification and therapeutic intervention.

The integration of scRNA-seq and spatial transcriptomics has emerged as an indispensable paradigm for dissecting the cellular architecture and molecular geography of solid tumors. While scRNA-seq provides unparalleled resolution for deconvolving cellular diversity and identifying rare subpopulations, it inherently sacrifices spatial context—a limitation that spatial transcriptomics overcomes by preserving the structural relationships and neighborhood interactions that define tissue function [32]. In the present study, the convergence of these technologies across three independent RCC cohorts enabled us to identify eight major cell populations and, critically, to localize *LMX1B* expression to specific epithelial regions within the tissue architecture. The marked reduction of *LMX1B* in tumor-derived epithelial cells relative to their normal counterparts, corroborated across qRT-PCR, TCGA, and CCLE datasets, underscores the fidelity of our integrative approach and highlights how single-cell and spatial platforms can collectively pinpoint candidate biomarkers that would be obscured in bulk transcriptomic analyses. This methodological framework aligns with the broader mission of the single-cell and spatial transcriptomics field: to move beyond cataloging cellular constituents toward understanding the spatially organized molecular programs that orchestrate tumor behavior.

Our CellChat-based ligand-receptor interaction analysis revealed that *LMX1B*-Epi cells exhibited the highest number of significant ligand-receptor interactions among epithelial cell populations., engaging in extensive bidirectional communication with fibroblasts, endothelial cells, myeloid cells, and T/NK cells [33]. The outgoing *VEGFA-VEGFR1/VEGFR2*, SPP1-CD44/ITGAV+ITGB1, and GDF15-TGFBR2 signaling axes, together with incoming *TNF*-*TNFRSF1A* and *PTN*-*SDC4*/*NCL* signals, position *LMX1B*-Epi as both active signal emitters and receivers—a dual role that is characteristic of cellular populations that organize tissue architecture. The spatial deconvolution and mistyR colocalization analyses further demonstrated that *LMX1B*-Epi cells showed close spatial proximity to fibroblasts, suggesting potential interactions between epithelial tumor cells and stromal components within the RCC microenvironment, suggesting that *LMX1B* loss may drive epithelial cells toward a more invasive, stroma-interacting phenotype [31]. These findings resonate with emerging spatial transcriptomic studies across diverse solid tumors, which have consistently demonstrated that the spatial distribution and neighborhood composition of tumor cells are as consequential as their intrinsic transcriptional states in determining malignant potential [32].

The hdWGCNA analysis illuminated the modular transcriptional architecture governed by *LMX1B* in RCC epithelial cells, identifying five co-expression modules with distinct functional identities [34]. Notably, the M5 mitochondrial ATP synthesis module, featuring genes such as *ATP5MC2, ATP5F1E*, and *ATP5F1D*, exhibited significant differential activation between normal and tumor *LMX1B*-Epi, with tumor cells displaying elevated mitochondrial dysfunction signatures. This observation is particularly salient given the central role of metabolic reprogramming in RCC pathogenesis, where the loss of *VHL*-driven pseudohypoxic signaling rewires cellular metabolism toward aerobic glycolysis and impairs oxidative phosphorylation. The enrichment of M1 (stress response) and M3 (acute-phase response) modules in tumor *LMX1B*-Epi further suggests that *LMX1B* downregulation sensitizes epithelial cells to pathophysiological stress while simultaneously compromising their capacity for adaptive metabolic responses. Collectively, these module-level findings provide a transcriptional blueprint for understanding how *LMX1B* loss predisposes renal epithelial cells toward malignant transformation.

Mechanistically, we established that *LMX1B* transcriptionally activates *GDF15* through direct binding to a conserved motif within its promoter region on chromosome 19p13.11. This finding positions *GDF15* as a bona fide downstream effector of *LMX1B*-mediated tumor suppression. The functional validation of *GDF15 in vitro* and *in vivo*—where its knockdown enhanced proliferation, migration, and invasion while its overexpression suppressed these malignant phenotypes—confirms its tumor-suppressive role in the renal context. The subsequent identification of *ATP4B* as a *GDF15*-regulated mediator, validated through TCGA stratification, western blot, and comprehensive rescue experiments, completes the *LMX1B*–*GDF15*–*ATP4B* regulatory axis. The observation that *ATP4B* overexpression completely reversed the oncogenic effects of *GDF15* knockdown in both cellular and xenograft models underscores the hierarchical and non-redundant nature of this signaling cascade.

Furthermore, we observed that *GDF15* positively regulates *ATP4B* expression, thereby inhibiting malignant progression in RCC. *ATP4B* is best characterized as the β-subunit of the gastric H⁺/K⁺-ATPase complex, where it contributes to proton secretion and gastric acid homeostasis. Although its function in renal tissue remains less well defined, available evidence suggests that *ATP4B* is expressed in renal tubular compartments, indicating potential roles beyond gastric physiology. Given the involvement of H⁺/K⁺-ATPase-related mechanisms in epithelial ion transport and acid–base regulation, *ATP4B* may contribute to the maintenance of tubular homeostasis and local pH balance in the kidney. However, whether *ATP4B* directly regulates renal ion transport or tubular function requires further investigation. From a translational perspective, therapeutic modulation of *ATP4B* should be approached cautiously. Although restoring *ATP4B* activity may represent a strategy to enhance tumor suppression in RCC, potential effects on renal acid–base equilibrium, electrolyte handling, and tubular physiology need to be carefully evaluated. Future studies using kidney-specific models and comprehensive renal function assessments will be necessary to determine the safety profile of *ATP4B*-targeted interventions (Fig. S7).

The mitochondrial apoptosis pathway emerged as the critical execution mechanism downstream of the *LMX1B*–*GDF15*–*ATP4B* axis. *GDF15* knockdown reduced intracellular ROS levels [35^,^ 36], increased mitochondrial membrane potential, and suppressed the expression of pro-apoptotic Bax, cytochrome c, and cleaved caspase-3, accompanied by a diminished Bax/Bcl-2 ratio [37]. These findings are consistent with the established paradigm in which ROS serve as pivotal mediators of mitochondrial outer membrane permeabilization: elevated ROS trigger oxidative stress, which activates the mitochondrial permeability transition pore, leading to membrane potential depolarization and the release of pro-apoptotic factors into the cytosol. The self-sustaining cycle of ROS generation and mitochondrial dysfunction creates a feed-forward loop that amplifies apoptotic signaling [38]. In this context, *GDF15* appears to function as a gatekeeper of mitochondrial integrity in RCC cells, with its *LMX1B*-dependent expression maintaining the ROS-MMP balance necessary for Bax/Bcl-2-caspase-3 cascade activation [39^,^ 40]. The immunohistochemical confirmation of reduced cleaved caspase-3 in *GDF15*-knockdown xenografts further validates the *in vivo* relevance of this mitochondrial apoptosis axis.

From a translational perspective, our findings position *LMX1B* as a compelling prognostic biomarker and therapeutic target for RCC. The inverse correlation between *LMX1B* expression and both tumor grade and lymphatic metastasis, observed across multi-platform datasets, suggests that *LMX1B* loss may serve as an early indicator of malignant progression. Moreover, the identification of a druggable axis—wherein *LMX1B* transcriptionally controls *GDF15*, which in turn regulates *ATP4B* and mitochondrial apoptosis—provides multiple nodes for therapeutic intervention. Small molecules that stabilize or activate *LMX1B*, recombinant *GDF15*, or *ATP4B*-targeted strategies could potentially restore apoptotic competence in *LMX1B*-deficient RCC cells. The context-dependent role of *GDF15* in cancer biology necessitates cautious interpretation. Although *GDF15* has been reported to promote tumor progression in prostate and pancreatic cancers, while exhibiting tumor-suppressive effects in colorectal cancer and RCC, the underlying basis for these divergent functions remains incompletely understood. One possible explanation is that *GDF15* signaling is highly dependent on cellular context, including receptor availability and downstream pathway activation. For example, *GDF15* can signal through the GFRAL–RET pathway in specific physiological and metabolic contexts [20^,^ 21], whereas TGFBR2-mediated signaling has been implicated in tumor-associated responses in peripheral tissues. In addition, differences in tumor microenvironment composition, including immune and stromal cell interactions, as well as metabolic states of distinct cancer types, may influence the biological consequences of *GDF15* activation. Therefore, therapeutic strategies targeting the *LMX1B*–*GDF15*–*ATP4B* axis should consider the tissue-specific signaling landscape and require further validation in RCC-specific models.

Nevertheless, the therapeutic targeting of *LMX1B* presents several important translational challenges. As a nuclear transcription factor, *LMX1B* lacks intrinsic enzymatic activity and may not be readily accessible to conventional small-molecule inhibitors. Furthermore, given its physiological roles in kidney development and tissue homeostasis, systemic inhibition of *LMX1B* may potentially result in unintended effects on normal tissues. Therefore, future therapeutic strategies may require approaches that selectively modulate *LMX1B* downstream effectors, disrupt specific transcriptional interactions, or employ targeted delivery systems to minimize off-target toxicity.

In addition, whether the *LMX1B*–*GDF15*–*ATP4B* axis influences sensitivity to currently available RCC therapies remains an important area for investigation. Given its involvement in epithelial differentiation, mitochondrial regulation, and tumor microenvironmental organization, this axis may potentially affect responses to tyrosine kinase inhibitors, mTOR inhibitors, or immune checkpoint blockade. Future studies integrating *LMX1B*–*GDF15*–*ATP4B* expression profiles with clinical treatment cohorts will be necessary to determine whether this pathway can serve as a predictive biomarker for therapeutic response or guide patient stratification.

Several limitations of this study warrant consideration. First, while our scRNA-seq and spatial transcriptomic analyses were conducted across three independent cohorts, the sample size for spatial transcriptomics was limited to four FFPE samples, which may constrain the generalizability of our spatial findings. Second, although *LMX1B* downregulation was consistently observed across multiple RCC datasets, the molecular mechanisms responsible for its reduced expression remain unclear. Potential contributors may include genetic alterations such as copy-number changes or somatic mutations, epigenetic regulation including promoter hypermethylation, and post-transcriptional regulation mediated by microRNAs. Future studies integrating genomic, epigenomic, and transcriptomic analyses will be required to elucidate the mechanisms underlying *LMX1B* silencing in RCC. Third, the functional role of *GDF15* may evolve during tumor progression, potentially transitioning from tumor suppression in early-stage disease to tumor promotion in advanced metastatic settings—a temporal dynamic that our current experimental design does not capture [22^,^ 39^,^ 41]. Fourth, while *ATP4B* has been primarily characterized in gastric acid secretion, its renal tubular function and the potential nephrotoxicity of *ATP4B*-targeted therapies require careful evaluation. Finally, the clinical translation of our findings will require validation in larger, multi-institutional cohorts and the development of preclinical models that more faithfully recapitulate the human RCC TME, including patient-derived organoids and humanized immune system mouse models.

In summary, our integrative single-cell and spatial transcriptomic analyses, complemented by multidimensional functional validation, establish *LMX1B* as a tumor suppressor that governs TME organization and mitochondrial homeostasis in renal cell carcinoma through the *GDF15*–*ATP4B* axis. These findings not only illuminate the molecular mechanisms underlying RCC progression but also identify *LMX1B* as a promising prognostic indicator and therapeutic target, offering a foundation for the development of precision oncology strategies in this lethal malignancy.

## Ethics statement

The Ethics Committee of the First Affiliated Hospital of Zhengzhou University approved the study design with the informed consent of all study participants. (2022-KY-1089-001)

## Funding

This work supported by the 10.13039/501100001809National Natural Science Foundation of China (grant number: 82172564, 82303032, 82573338).

## Generative AI statement

The author(s) declare that no Generative AI was used in the creation of this manuscript.

## CRediT authorship contribution statement

**Yu Liu:** Writing – original draft, Visualization, Methodology, Investigation, Formal analysis, Data curation. **Liang Song:** Writing – original draft, Visualization, Methodology, Investigation, Formal analysis, Data curation. **Yuankang Feng:** Writing – original draft, Validation, Supervision, Methodology, Investigation, Formal analysis, Data curation. **Songxin Zhang:** Investigation, Formal analysis. **Xu Han:** Investigation. **Youzhi Tan:** Data curation. **Ningyang Li:** Data curation. **Yingjin Wang:** Writing – review & editing. **Shaoliang Sun:** Writing – review & editing. **Jingwei Gao:** Visualization. **Long Zhang:** Writing – review & editing, Supervision, Project administration, Conceptualization. **Zhankui Jia:** Writing – review & editing, Project administration, Conceptualization. **Jinjian Yang:** Writing – review & editing, Conceptualization.

## Declaration of competing interest

The authors declare that they have no known competing financial interests or personal relationships that could have appeared to influence the work reported in this paper.

## Data Availability

All data analyzed in this study are publicly available. The renal cell carcinoma (RCC) single-cell RNA sequencing (scRNA-seq) datasets GSE230358, GSE230359, and GSE230360 were obtained from the Gene Expression Omnibus (GEO) database. The spatial transcriptomics data (GSE175540) were also retrieved from GEO. Bulk RNA-sequencing data for the TCGA-KIRC (Kidney Renal Clear Cell Carcinoma) cohort were downloaded from the UCSC Xena platform. The independent validation dataset GSE12008 was retrieved from GEO. The Cancer Cell Line Encyclopedia (CCLE) database was queried for *LMX1B* mRNA expression across RCC cell lines. Transcription factor binding predictions were performed using the JASPAR database. Chromosomal localization and promoter architecture analysis were conducted using the National Center for Biotechnology Information (NCBI) Reference Sequence database. Any additional data supporting the conclusions of this study are available from the corresponding author upon reasonable request.
