# Supplementary material for: Integrative single-cell and spatial transcriptomic analyses identify LMX1B as a tumor suppressor orchestrating the GDF15–ATP4B axis in renal cell carcinoma
Source: Transl Oncol. 2026 Sep 18;73:103049. doi: 10.1016/j.tranon.2026.103049 (PMC13625895; doi:10.1016/j.tranon.2026.103049)
Supplement: Supplementary file 1 [file mmc1.docx]

Supplementary Material

**Integrative single-cell and spatial transcriptomic analyses identify LMX1B as a tumor suppressor orchestrating the GDF15–ATP4B axis in renal cell carcinoma**

Yu Liu1†, Liang Song1†, Yuankang Feng1,2†, Songxin Zhang1, Xu Han1, Youzhi Tan1, Ningyang Li1, Yingjin Wang1, Shaoliang Sun1, Jingwei Gao1, Long Zhang1***, Zhankui Jia1**, Jinjian Yang1*

*** Correspondence:** Corresponding Author: [yangjinjian2011@126.com](mailto:yangjinjian2011@126.com),jiazhankui@126.com,zhanglongtbz@163.com

**Files included in Supplementary information**

**Supplementary Table 1. Cell lines, antibodies and other reagents and resources**

**Supplementary Table 2. Target sequences used to silence GDF15 expression**

**Supplementary Table 3. Sequence information of primers for RT-qPCR**

**Supplementary Table 4. The DEGs of top 20 KEGG pathways.**

**Supplementary Figure 1. The expression of LMX1B in ccRCC tissues and cells.**

**Supplementary Figure 2. GDF15 inhibited proliferation and invasion of RCC cells *in vitro*.**

**Supplementary Figure 3. ATP4B is the downstream target of GDF15.**

**Supplementary Figure 4. ATP4B inhibited proliferation and invasion of RCC cells *in vitro*.**

**Supplementary Figure 5. ATP4B could regulate cell apoptosis.**

**1. Supplementary Tables**

**Supplementary Table 1. Cell lines, antibodies and other reagents and resources**

| **Cell lines** | **Cat.** | **Company** |
| --- | --- | --- |
| HK-2 (Human renal proximal tubule cells) | [GNHu47](https://www.cellbank.org.cn/search-detail.php?id=829) | [The Chinese National Stem Cell Resource Center](https://www.sciencedirect.com/science/article/pii/S1873506120302865) |
| 769-P (Human renal cell adenocarcinoma cells) | [TCHu2](https://www.cellbank.org.cn/search-detail.php?id=337)15 | [The Chinese National Stem Cell Resource Center](https://www.sciencedirect.com/science/article/pii/S1873506120302865) |
| 786-O (Human renal clear cell adenocarcinoma cells) | [TCHu186](https://www.cellbank.org.cn/search-detail.php?id=89) | [The Chinese National Stem Cell Resource Center](https://www.sciencedirect.com/science/article/pii/S1873506120302865) |
| ACHN (Human renal cell adenocarcinoma cells) | [TCHu199](https://www.cellbank.org.cn/search-detail.php?id=337) | [The Chinese National Stem Cell Resource Center](https://www.sciencedirect.com/science/article/pii/S1873506120302865) |
| **Antibodies** | **Cat.** | **Company** |
| LMX1B WB 1:1000 | ab259926 | Abcam |
| GDF15 WB 1:5000 | ab180929 | Abcam |
| Bax WB 1:1000 | 41162 | Cell Signaling Technology |
| Bcl2 WB 1:1000 | 15071 | Cell Signaling Technology |
| Cyt C  WB 1:5000 | ab133504 | Abcam |
| caspase3 WB 1:1000 | 9662 | Cell Signaling Technology |
| cleaved caspase3  WB 1:1000 | 9664 | Cell Signaling Technology |
| cleaved caspase3 IHC 1:500 | GB11532-100 | Servicebio |
| ATP4B WB 1:5000 | ab186749 | Abcam |
| β-Actin WB 1:5000 | ab8227 | Abcam |
| Dylight 800-Goat Anti-Rabbit IgG WB 1:10000 | A23910 | Abbkin |
| Dylight 800-Goat Anti-Mouse IgG WB 1:50000 | A23920 | Abbkin |

**Supplementary Table 2. Target sequences used to silence GDF15 expression**

| **Gene** | **Sequence** |
| --- | --- |
| sh#1*-GDF15* | AACCTGCACAGCCATGCCCGG |
| sh#2*-GDF15* | AACTCAGGACGGTGAATGGCT |
| sh#3*-GDF15* | GGATACTCACGCCAGAAGTGCG |

**Supplementary Table 3. Sequence information of primers for RT-qPCR**

| **Species** | **Gene** | **Forward (5’-3’)** | **Reverse (5’-3’)** |
| --- | --- | --- | --- |
| Human | GAPDH | TGACATCAAGAAGGTGGTGAAGCAG | GTGTCGCTGTTGAAGTCAGAGGAG |
| Human | LMX1B | TGGACGGCATCAAGATGGAGGAG | GACTCGTTGACTCGCATCAGGAAG |
| Human | GDF15 | TCAGGACGGTGAATGGCTCTCAG | TCGGAATCTGGAGTCTTCGGAGTG |
| Human | ATP4B | TACCCTCCCAACGGCACCTTC | GCACACGATGGCGACCTCAG |

**Supplementary Table 4. The DEGs of top 20 KEGG pathways.**

| **Category** | **Term** | **Description** | **Log(q-value)** | **Symbols** |
| --- | --- | --- | --- | --- |
| Reactome Gene Sets | R-HSA-382551 | Transport of small molecules | -6.875233447 | ASIC1, APOC3, AQP6, ATP4B, ATP6V1B1, CLCNKA, CLCNKB, HBA1, LCN2, PIP, SCNN1A, SLC4A1, SLC6A2, SLC9A2, SLC12A1, BSND, SLC22A8, SLC22A7, FGF21, ATP6V0A4, RHCG, FXYD4, SLC6A15, RHBG, SLC13A3, SLC4A9, SLC26A7, ATP6V1G3, SLC36A2, ATP6V0D2, ABCB5, SLC9A4, GABRA5, KCNJ13, HTR3B, CACNG2, KCNIP1, TMEM38A, KCNG3, LRRC52, UMOD, SLC38A8, HRG, CALCA, TNNI3, ERFE, SYNGR3 |
| Reactome Gene Sets | R-HSA-382551 | Transport of small molecules | -6.875233447 | ASIC1, APOC3, AQP6, ATP4B, ATP6V1B1, CLCNKA, CLCNKB, HBA1, LCN2, PIP, SCNN1A, SLC4A1, SLC6A2, SLC9A2, SLC12A1, BSND, SLC22A8, SLC22A7, FGF21, ATP6V0A4, RHCG, FXYD4, SLC6A15, RHBG, SLC13A3, SLC4A9, SLC26A7, ATP6V1G3, SLC36A2, ATP6V0D2, ABCB5, SLC9A4 |
| GO Biological Processes | GO:0098660 | inorganic ion transmembrane transport | -6.875233447 | ASIC1, AQP6, ATP4B, ATP6V1B1, CLCNKA, CLCNKB, GABRA5, KCNJ13, SCNN1A, SLC4A1, SLC6A2, SLC9A2, SLC12A1, BSND, HTR3B, CACNG2, KCNIP1, ATP6V0A4, RHCG, FXYD4, SLC6A15, RHBG, TMEM38A, SLC4A9, SLC26A7, ATP6V1G3, SLC36A2, KCNG3, ATP6V0D2, SLC9A4, LRRC52 |
| GO Biological Processes | GO:0055067 | monovalent inorganic cation homeostasis | -4.459556575 | ATP4B, ATP6V1B1, SCNN1A, SLC4A1, SLC9A2, SLC12A1, UMOD, ATP6V0A4, RHCG, RHBG, SLC4A9, ATP6V0D2, SLC9A4 |
| GO Biological Processes | GO:0006814 | sodium ion transport | -4.428696796 | ASIC1, ATP4B, ATP6V1B1, CLCNKB, SCNN1A, SLC6A2, SLC9A2, SLC12A1, UMOD, SLC6A15, SLC13A3, SLC4A9, SLC38A8, SLC9A4 |
| GO Biological Processes | GO:0098662 | inorganic cation transmembrane transport | -4.12956017 | ASIC1, ATP4B, ATP6V1B1, KCNJ13, SCNN1A, SLC6A2, SLC9A2, SLC12A1, HTR3B, CACNG2, KCNIP1, ATP6V0A4, RHCG, FXYD4, SLC6A15, RHBG, TMEM38A, SLC4A9, ATP6V1G3, SLC36A2, KCNG3, ATP6V0D2, SLC9A4, LRRC52 |
| GO Biological Processes | GO:0098655 | cation transmembrane transport | -3.412586138 | ASIC1, ATP4B, ATP6V1B1, KCNJ13, SCNN1A, SLC6A2, SLC9A2, SLC12A1, HTR3B, CACNG2, KCNIP1, ATP6V0A4, RHCG, FXYD4, SLC6A15, RHBG, TMEM38A, SLC4A9, ATP6V1G3, SLC36A2, KCNG3, ATP6V0D2, SLC9A4, LRRC52 |
| GO Biological Processes | GO:0030001 | metal ion transport | -3.390914405 | ASIC1, ATP4B, ATP6V1B1, CLCNKB, HRG, KCNJ13, LCN2, SCNN1A, SLC6A2, SLC9A2, SLC12A1, UMOD, CACNG2, KCNIP1, FXYD4, SLC6A15, SLC13A3, TMEM38A, SLC4A9, SLC38A8, KCNG3, SLC9A4, LRRC52 |
| GO Biological Processes | GO:0030004 | cellular monovalent inorganic cation homeostasis | -3.260945885 | ATP4B, ATP6V1B1, SCNN1A, SLC4A1, SLC9A2, UMOD, ATP6V0A4, SLC4A9, ATP6V0D2, SLC9A4 |
| GO Biological Processes | GO:0006885 | regulation of pH | -2.831710027 | ATP4B, ATP6V1B1, SLC4A1, SLC9A2, ATP6V0A4, RHCG, SLC4A9, ATP6V0D2, SLC9A4 |
| GO Biological Processes | GO:0071805 | potassium ion transmembrane transport | -2.016208466 | ATP4B, KCNJ13, SLC9A2, SLC12A1, KCNIP1, FXYD4, TMEM38A, KCNG3, SLC9A4, LRRC52 |
| GO Biological Processes | GO:0035725 | sodium ion transmembrane transport | -2.016208466 | ASIC1, ATP4B, SCNN1A, SLC6A2, SLC9A2, SLC12A1, SLC6A15, SLC4A9, SLC9A4 |
| GO Biological Processes | GO:0006813 | potassium ion transport | -1.947541468 | ATP4B, KCNJ13, SLC9A2, SLC12A1, KCNIP1, FXYD4, TMEM38A, KCNG3, SLC9A4, LRRC52 |
| GO Biological Processes | GO:0055080 | cation homeostasis | -1.866075043 | ATP4B, ATP6V1B1, CALCA, LCN2, SCNN1A, SLC4A1, SLC9A2,  SLC12A1, TNNI3, UMOD, ATP6V0A4, RHCG, RHBG, SLC4A9, ERFE, ATP6V0D2, SLC9A4 |
| GO Biological Processes | GO:0098771 | inorganic ion homeostasis | -1.775571876 | ATP4B, ATP6V1B1, CALCA, LCN2, SCNN1A, SLC4A1, SLC9A2, SLC12A1, TNNI3, UMOD, ATP6V0A4, RHCG, RHBG, SLC4A9, ERFE, ATP6V0D2, SLC9A4 |
| GO Biological Processes | GO:0051453 | regulation of intracellular pH | -1.775571876 | ATP6V1B1, SLC4A1, SLC9A2, ATP6V0A4, SLC4A9, ATP6V0D2, SLC9A4 |
| GO Biological Processes | GO:0006873 | cellular ion homeostasis | -1.721730673 | ATP4B, ATP6V1B1, CALCA, LCN2, SCNN1A, SLC4A1, SLC9A2, TNNI3, UMOD, ATP6V0A4, RHCG, SLC4A9, ERFE, ATP6V0D2, SLC9A4 |
| GO Biological Processes | GO:0050801 | ion homeostasis | -1.720923682 | ATP4B, ATP6V1B1, CALCA, LCN2, SCNN1A, SLC4A1, SLC9A2, SLC12A1, TNNI3, UMOD, ATP6V0A4, RHCG, RHBG, SLC4A9, ERFE, ATP6V0D2, SLC9A4 |
| GO Biological Processes | GO:0030641 | regulation of cellular pH | -1.69791599 | ATP6V1B1, SLC4A1, SLC9A2, ATP6V0A4, SLC4A9, ATP6V0D2, SLC9A4 |
| GO Biological Processes | GO:0030003 | Cellular cation homeostasis | -1.454363448 | ATP4B, ATP6V1B1, CALCA, LCN2, SCNN1A, SLC4A1, SLC9A2, TNNI3, UMOD, ATP6V0A4, SLC4A9, ERFE, ATP6V0D2, SLC9A4 |

**Supplementary Figure 1. Cell-cell communication analysis and spatial deconvolution of renal cell carcinoma.**

(A) Circle plots showing the number of interactions (top) and interaction weight/strength (bottom) between different cell types in RCC, including B/Plasma cells, Endothelial cells, LMX1B-Epi, Fibroblasts, Mast cells, Myeloid cells, Prolif, and T/NK cells. Edge thickness and color intensity represent the magnitude of interactions.(B) Dot plot showing significant ligand-receptor pairs from LMX1B-associated epithelial cells (LMX1B-Epi) to other cell types. The dot size represents the p-value, and the color intensity indicates the communication probability.(C) Dot plot showing significant ligand-receptor pairs from other cell types to LMX1B-associated epithelial cells (LMX1B-Epi). The dot size represents the p-value, and the color intensity indicates the communication probability.(D) Hematoxylin and eosin (H&E) staining image of the ffpe_c_2 spatial transcriptomics (ST) sample.(E) Seurat cluster spatial map of the ffpe_c_2 ST sample, with clusters labeled 0–13 and color intensity indicating spatial density from minimum (blue) to maximum (red).(F) RCTD deconvolution results showing the spatial distribution and proportion of each cell type in the ffpe_c_2 sample, including B/Plasma cells, Endothelial cells, Fibroblasts, LMX1B-Epi, Mast cells, Myeloid, Prolif, and T_NK cells. Color intensity indicates cell type abundance from minimum (blue/purple) to maximum (yellow/red).(G) mistyR spatial colocalization analysis showing the pairwise spatial correlation between cell types across all ST samples (global). Top: Heatmap of spatial correlation coefficients. Bottom: Network plot highlighting significant spatial colocalization relationships.(H) mistyR spatial colocalization analysis showing the pairwise spatial correlation between cell types within the juxta_5 spatial context (5-spot radius). Top: Heatmap of spatial correlation coefficients. Bottom: Network plot highlighting significant spatial colocalization relationships.(I) mistyR spatial colocalization analysis showing the pairwise spatial correlation between cell types within the para_15 spatial context (15-spot radius). Top: Heatmap of spatial correlation coefficients. Bottom: Network plot highlighting significant spatial colocalization relationships.

**Supplementary Figure 2. Hierarchical differential weighted gene co-expression network analysis (hdWGCNA) of LMX1B-associated epithelial cells in renal cell carcinoma.**

(A) Selection of the optimal soft-thresholding power based on scale-free topology fit and connectivity analyses, identifying power = 7 as the optimal parameter.(B) Hierarchical clustering dendrogram of gene co-expression modules identified by hdWGCNA, revealing six modules (M1–M5).(C) Module eigengene bar plots showing the top hub genes with highest module membership (kME) for each of the five identified modules (M1–M5). The x-axis represents kME values.(D) UMAP visualizations of single cells colored by module eigengene scores for modules M1–M5 in epithelial cells. The color intensity indicates the level of module eigengene expression.(E) Network visualization of module eigengene relationships between normal and tumor LMX1B-associated epithelial cells. Node size represents module size, edge thickness indicates correlation strength, and node colors correspond to module identities (M1–M5). Module labels are indicated for each node.(F) UMAP visualization of all cells colored by average module expression (left) and percent of expressed genes (right), with module eigengene scores overlaid.(G) Dot plot showing Gene Ontology (GO) biological process enrichment analysis for modules M1–M5. The dot size represents the gene ratio (percent of module genes in the pathway), and the color intensity indicates the -log10(P-value) of enrichment significance. Key pathways include nucleotide-binding oligomerization domain (NOD)-like receptor signaling, stress-induced intrinsic apoptotic signaling, cellular respiration, mitochondrial ATP synthesis, aerobic electron transport, fibrinolysis, and blood coagulation.(H) Violin plots comparing module eigengene scores between normal and tumor LMX1B-associated epithelial cells for modules M1–M5. Statistical significance is indicated by asterisks (****P < 0.0001). The y-axis shows module eigengene score values.

**Supplementary Fig. 3. The expression of LMX1B in ccRCC tissues and cells.** (A) Correlation between transfection duration and LMX1B expression levels. Data are mean ± SEM (n = 3). one-way ANOVA with Dunnett's test (B) Quantification of EdU-positive cells after LMX1B overexpression. Data are mean ± SEM (n = 3). unpaired two-tailed Student's t-test. (C) GDF15 expression following LMX1B overexpression. Data are mean ± SEM (n = 3). unpaired two-tailed Student's t-test. n.s., not significant, *P < 0.05, **P < 0.01, ***P < 0.001,****P < 0.0001.

**Supplementary Fig. 4. GDF15 inhibited proliferation and invasion of RCC cells *in vitro*.** (A, B) Validation of GDF15 overexpression efficiency by qRT-PCR (A) and western blot (B). Data are mean ± SEM (n = 3). unpaired two-tailed Student's t-test. (C) Representative images (left) and quantification (right) of Transwell migration and invasion assays in RCC cells with GDF15 overexpression. Data are mean ± SEM (n = 3). unpaired two-tailed Student's t-test. (D) Cell proliferation measured by CCK-8 assay in RCC cells with GDF15 overexpression. Data are mean ± SEM (n = 3). two-way repeated measures ANOVA with Tukey's multiple comparisons test .(E) Representative images and quantification of colony formation assays in RCC cells with GDF15 overexpression. Data are mean ± SEM (n = 3). unpaired two-tailed Student's t-test.*P < 0.05, **P < 0.01, ***P < 0.001,****P < 0.0001.

**Supplementary Fig. 5. GDF15 affects the cell proliferation.** (A, B) Representative images (left) and quantification (right) of EdU proliferation assays in RCC cells transfected with sh-NC, sh-GDF15#1, or sh-GDF15#2. Data are mean ± SEM (n = 3). one-way ANOVA with Dunnett's multiple comparisons test (vs. sh-NC).*P < 0.05, **P < 0.01, ***P < 0.001,****P < 0.0001.

**Supplementary Fig. 6. GDF15 could regulate cell apoptosis.** (A)Quantitative assessment of reactive oxygen species (ROS) levels following GDF15 knockdown. Data are mean ± SEM (n = 3). one-way ANOVA with Dunnett's multiple comparisons test (vs. sh-NC). (B, C) Quantification of apoptotic pathway protein levels after GDF15 knockdown. Data are mean ± SEM (n = 3). one-way ANOVA with Dunnett's multiple comparisons test (vs. sh-NC). (D, E) Representative immunohistochemistry (IHC) images (D) and quantification (E) of cleaved caspase-3 levels after GDF15 knockdown. Data are mean ± SEM (n = 3).one-way ANOVA with Dunnett's multiple comparisons test (vs. sh-NC). (F, G) Western blot analysis and quantification of apoptotic pathway proteins after GDF15 knockdown and ATP4B overexpression. Data are mean ± SEM (n = 3). two-way ANOVA with Tukey's multiple comparisons test. (H) ATP4B mRNA expression in normal (n = 72) and primary tumor (n = 533) tissues from the TCGA-KIRC cohort, analyzed using the UALCAN database. n.s., not significant (P > 0.05); *P < 0.05, **P < 0.01, ***P < 0.001, ****P < 0.0001

**Supplementary Fig. 7. ATP4B inhibited proliferation and invasion of RCC cells *in vitro, also* regulate cell apoptosis.** (A, B) Validation of ATP4B overexpression efficiency by qRT-PCR and western blot in 786-O and ACHN cells. Data are mean ± SEM (n = 3). unpaired two-tailed Student's t-test. (C) Cell proliferation measured by CCK-8 assay in 786-O and ACHN cells transfected with empty vector or ATP4B OE. Data are mean ± SEM (n = 3). two-way repeated measures ANOVA .(D) Representative images and quantification of Transwell migration and invasion assays in 786-O and ACHN cells. Data are mean ± SEM (n = 3). unpaired two-tailed Student's t-test. (E) Representative images and quantification of colony formation assays in 786-O and ACHN cells. Data are mean ± SEM (n = 3). unpaired two-tailed Student's t-test. (F–H) Western blot analysis of apoptosis-related proteins after ATP4B overexpression. (F) Representative images; (G, H) Quantification of cleaved caspase-3 and Cytochrome c levels. unpaired two-tailed Student's t-test. n.s., not significant (P > 0.05); *P < 0.05, **P < 0.01, ***P < 0.001, ****P < 0.0001
